# Pyrazole‐promoted synthesis of pyrrolo[3,4‐c] quinoline-1,3‐diones in a novel diketene-based reaction

**DOI:** 10.3389/fchem.2023.1219986

**Published:** 2023-09-26

**Authors:** Atieh Rezvanian, Zahra Esfandsar

**Affiliations:** Department of Organic Chemistry, Faculty of Chemistry, Alzahra University, Tehran, Iran

**Keywords:** pyrrolo[3,4-c]quinolones, pyrazole, diketene, heterocycles, green solvent, multicomponent reaction

## Abstract

We describe the first classic example of green synthesis of pyrrolo[3,4-c]quinolones scaffolds by catalyst-free unusual reaction of diketene, isatin, and primary amines in ethanol in the presence of pyrazole as a promoter for 4 h. The whole structure of the new product was confirmed by X-ray analysis. The overall transformation involves the cleavage and generation of multiple carbon-nitrogen and carbon-carbon bonds. This report represents a simple and straightforward approach for the synthesis of pyrrolo[3,4-c]quinoline-1,3-diones, which has significant advantages like readily available precursors, non-use of toxic solvent, operational simplicity, mild conditions, good atom economy, and excellent yields; therefore it provides a green and sustainable strategy for access to a range of interesting *N*-containing heterocyclic compounds in medicinal and organic chemistry.

## Graphical Abstract

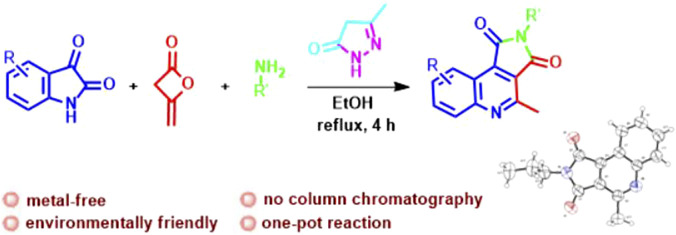

## 1 Introduction

Quinolines have received lots of attention from biologists and chemists as they are significant elements in the synthesis of dyes, fragrances, and natural products with biological activities (Michael, 2001; Michael, 2002; Michael, 2003; Michael, 2004; Michael, 2005; Michael, 2007; Michael, 2008; Isaac-Márquez et al., 2010; Cai et al., 2011; Russ et al., 2012). In pharmaceuticals, they have been outlined as, antibiotic (Mahamoud et al., 2006), anticancer (Insuasty et al., 2013; Sun et al., 2013), anti-inflammatory (Leatham et al., 1983), antimalarial (Nasveld and Kitchener, 2005), antihypertensive (Muruganantham et al., 2004), anti-HIV (Strekowski et al., 1991; Wilson et al., 1992), inhibition of Platelet-derived growth factor (PDGF) (Maguire et al., 1994), and anti-tuberculosis (Lilienkampf et al., 2009) agents. In other words, pyrrolidones are also typical buildings in several important categories of bioactive compounds (Jouyban et al., 2010; Kato and Nagao, 2012). Molecules bearing a pyrrolidone structure, are used in dye-sensitized solar cells and several natural products with biologically activeties (Daly et al., 1999; Dewick, 2009; Ikai et al., 2012). For instance, arcyria rubin A and its derivatives show potent antiviral activities (KIM et al., 1995; Slater et al., 1995), antimicrobial (Mahboobi et al., 2006), and powerful protein kinase C inhibitors (Davis et al., 1992).

The merger of these outstanding heterocycles, pyrrolidone, and quinolone is promising classes of pharmaceutical frameworks with antifungal (Chen et al., 2004), anti-inflammatory (Kategaonkar et al., 2010), anticancer (Eswaran et al., 2010), anti-tuberculosis (Tseng et al., 2010), anti-Alzheimer (Tseng et al., 2009), anti-HIV, anti-hypertension, and anticancer activities (Camps et al., 2009; Sharma et al., 2018). They also have inhibitory activities versus hepatitis C virus (HCV) polymerase (Thomas and Tallman, 1981; Summa et al., 2009), ADAMTS-5 (A disintegrin and metalloproteinase with thrombospondin motifs 5) and ADAMTS-4 (A disintegrin and metalloproteinase with thrombospondin motifs 4) (Sharma et al., 2008; Cappelli et al., 2010).

**FIGURE 1:**
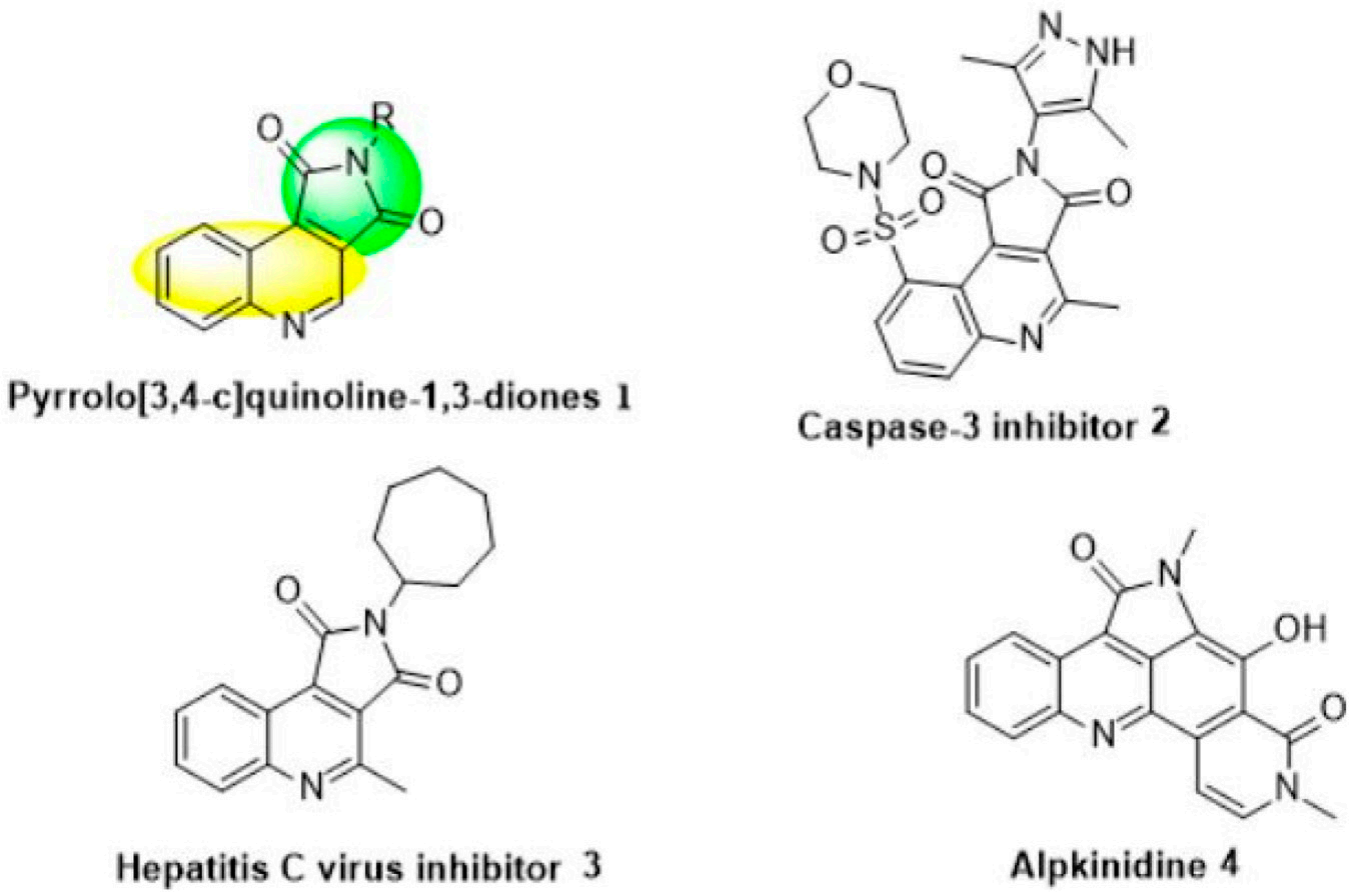
Bioactive pyrrolo[3,4-c]quinoline-2-ones.

In this regard, the pyrrolo[3,4-c]quinoline-1,3-dione segment (**1**) exhibits a perfect range of pharmacologically and biologically enjoyable activities (Figure 1) (Okun et al., 2006a; Okun et al., 2006b; Segura-Cabrera et al., 2011; Mollin et al., 2012). For example, pyrrolo[3,4-c]quinoline (**2**) is a potent inhibitor of caspase-3 (Kravchenko et al., 2005a), which plays a clef role in apoptosis (Porter and Jänicke, 1999; Hentze et al., 2003). Caspases are interesting goals for therapeutic intervention in neurodegenerative, cardiovascular and metabolic disorders (Lockshin et al., 1998; Lockshin and Zakeri, 2004). In particular, caspase-3 inhibitors have been reported as powerful hepatoprotectants (Lockshin et al., 1998; Hoglen et al., 2001; Segawa et al., 2001; Lockshin and Zakeri, 2004; Meki et al., 2004), cardioprotectants (Chapman et al., 2002; Isabel et al., 2003), and neuroprotectants (Scott et al., 2003). Also, compound (**3**) has inhibitory activity against HCV polymerase (Di Francesco et al., 2009). Furthermore, alpkinidine (**4**) has shown potent therapeutic efficacy *in vivo* in HCT-116-bearing mice (Valeriote et al., 2012).

Because of broad applications in medicinal chemistry, the synthesis of these fused interesting heterocycles has specific importance to the pharmaceutical and organic chemists. Newly, there has been increasing attentiveness in the construction of pyrrole-fused-quinolines, and various procedures have been reported. Main synthetic approaches include Lewis acid-catalyzed electrophilic cyclization (Aggarwal et al., 2012), copper (Kiruthika et al., 2014) and palladium-catalyzed (Chai and Lautens, 2009; Shukla et al., 2012; Kiruthika et al., 2014) reactions, DDQ-mediated intramolecular cyclization (Wald et al., 1980), allene-based reaction cascades (Baumann and Baxendale, 2015), photo substituted reactions and flash vacuum pyrolysis. Although the majority of synthetic plans were applied for the synthesis of pyrrolo[3,2-c]quinoline and pyrrolo[1,2-a]quinoline analogs. Few synthetic relate have been released on the synthesis of pyrrolo[3,4-c]quinolones, and only multi-step synthetic methods are known to date.

**SCHEME 1:**
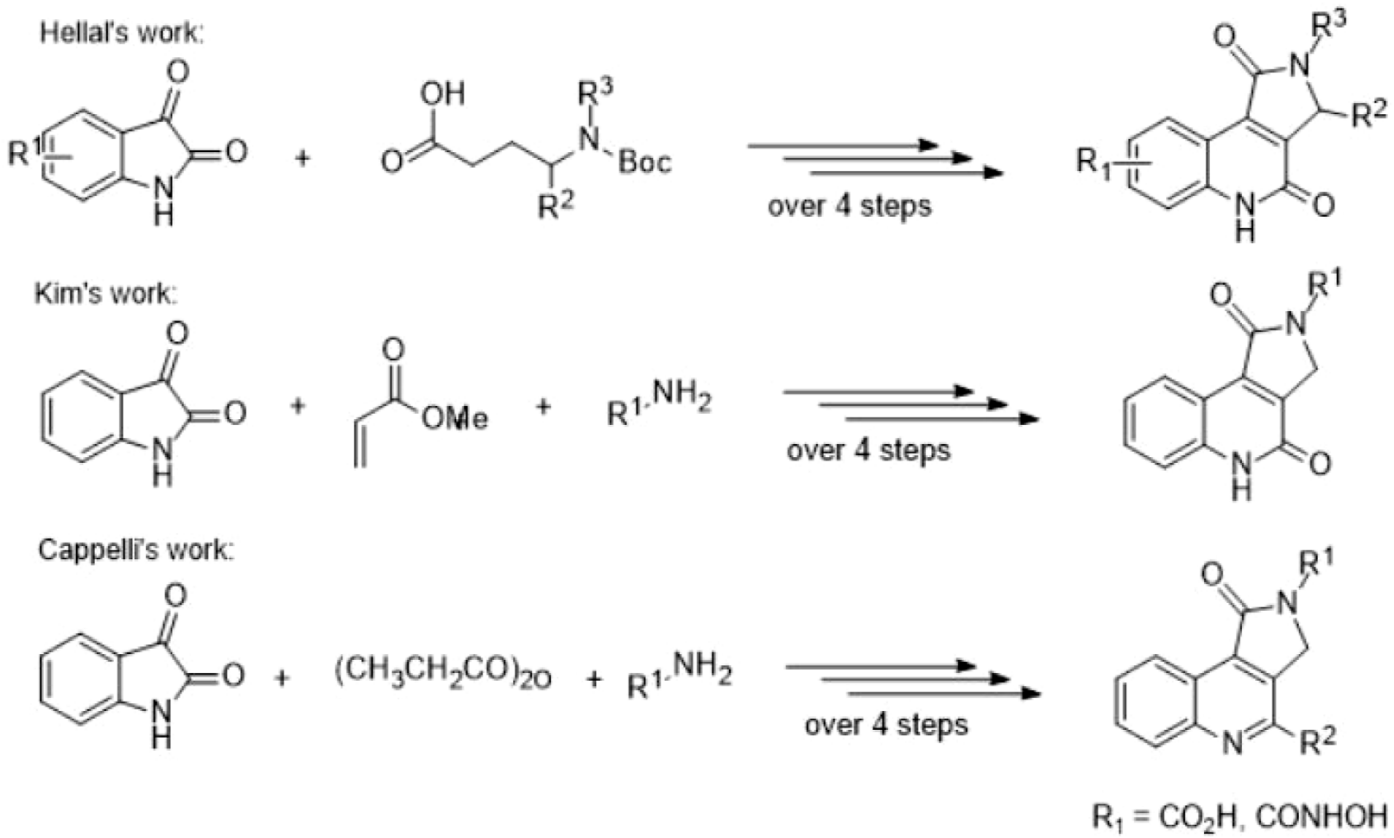
Multi-step; synthesis of pyrrolo[3,4-c]quinoline derivatives.

In this matter, there are notable examples based on the cyclo condensation of b-keto amides and 2-amino-5-fluorophenyl glyoxylic acid (Ivachtchenko et al., 2003), Pfitzinger reaction (Mortoni et al., 2004; Kravchenko et al., 2005b), the one-pot two-component method by DMAP-catalyzed (Avula et al., 2013), the BF_3_Et_2_O-catalyzed isocyanide-based cycloaddition reaction (Li et al., 2013), and microwave-assisted reaction methods (Xia et al., 2014)^,^ which all are multi-step reactions (Scheme 1). However, these procedures are limited by low yields, harsh reaction conditions, the long reaction time, and their complexity.

Multicomponent reactions (MCRs) have become increasingly popular as a simple and powerful tool for the rapid formation of new scaffolds from simple starting materials with structural diversity and molecular complexity in a convergent manner (Mashayekh and Shiri, 2019; Sedighian et al., 2021). MCRs are one-pot strategies exploiting three or more simple substrates where most of the reactant atoms are incorporated into the final desired product (Chen et al., 2017; Kurhade et al., 2019; Shiri and Aboonajmi, 2020; Yavari and Safaei, 2020). In comparison to the traditional multistep sequential assembly of target compounds, MCRs manifest several advantages including easy handling, selective bond formation, time-saving, high atom economy, fewer purification steps and structural variability (Younus et al., 2021; Shiri et al., 2022).

Due to our experience and interest in the synthesis of novel heterocycles, we became engrossed in how Knoevenagel product obtained from isatine and pyrazole could be *in situ* trapped by keto amides resulting from diketene and primary amines to give a heterocycle product. We considered the utilization of diketene as starting material and reagent because it is extensively used for the generation for a diverse range of different heterocycles. For this purpose, in continuation of our successive attempts towards the synthesis of heterocycles by multicomponent strategies, (Rezvanian et al., 2018a; Talaei et al., 2018; Rezvanian et al., 2020a; Rezvanian et al., 2020b), especially using diketene reactions (Alizadeh et al., 2012; Rezvanian, 2015; Rezvanian, 2016; Rezvanian et al., 2017; Rezvanian et al., 2018b; Rezvanian et al., 2019; Rezvanian et al., 2020c; Rezvanian et al., 2020d; Rezvanian et al., 2020e; Rezvanian et al., 2021a; Rezvanian et al., 2021b), we herein explain an efficient approach to synthesize pyrrolo[3,4-c]quinoline-1,3-diones **7** from the reaction of isatin, diketene, and primary amines based on the unique reactivity of pyrazole as a promoter in high yields (Scheme 3).

## 2 Experimental section

### 2.1 Instrumentation, analyses, and starting materials

The diketene, various amines, hydrazine, Hydrate, ethyl acetoacetate, and isatines were obtained from commercial sources with high purity. The ^1^H NMR and ^13^C NMR spectra were run on a Bruker spectrophotometer at 300/500 and 75/125 MHz respectively. Coupling constants are reported in Hz. All mass spectra were measured on a mass spectrometer (Agilent5973 Network) at the ionization potential of 70 Ev. The IR spectra were recorded by BRUKER TENSOR 27 FT-IR instrument.

### 2.2 General procedure for the synthesis of 3-methyl-pyrazole-5-one

Hydrazine hydrate 70% (2 mmol) was added to ethyl acetoacetate (1.4 mmol) and was treated at room temperature without solvent. After 30 min, the product was precipitate and filtered, and washed with a few drops of water, and pyrazole was obtained as a white crystal dried and used for further steps.

### 2.3 General procedure for the synthesis of structurally diverse pyrrolo[3,4-c]quinoline-1,3-diones 7

To a round-bottom flask (25 mL), the following were added; pyrazole (1.0 mmol), isatin **5** (1.0 mmol), diketene (1.0 mmol), primary amine **6** (1.0 mmol); and the reaction mixture was stirred at reflux for approximately 4 h and monitored by TLC until the substrates were wholly consumed. Upon the formation of the desired product **7**, the product was precipitated, filtered, and washed with a few drops of EtOH, and the target compound **7** was obtained as a yellow solid with excellent yield (73%–90%). Post separating product **7**, the reaction mixture was cooled to 20°C–25°C, and upon cooling the reaction mixture and evaporation of the solvent, the sediment solid was filtered and washed with ethanol, and finally the pyrazole was obtained again with 81% yield.

## 3 Results and discussion

**SCHEME 2:**
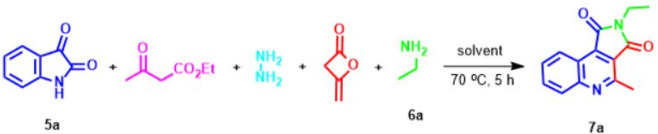
Synthesis of pyrrolo[3,4-c]quinoline-1,2-dione **7a**.

At the outset of our investigation, the reaction of hydrazine, ethyl acetoacetate, isatine **5**, diketene, and primary amine **6** in the lack of any catalyst at room temperature was designed. To study this new process, isatin **5a**, ethyl amine **6a**, and ethyl acetoacetate were selected as model reactions (Scheme 2). In this route, firstly, hydrazine (1 mmol), ethyl acetoacetate (1 mmol), and isatin **5a** (1 mmol) in ethanol (4 ml) were stirred at room temperature for 1 h, which afforded the Michael adduct **8a**. Next, ethylamine **6a** (1 mmol) and diketene (1 mmol) were added to the reaction mixture. The advance of the reaction was followed by TLC (1-6 ethyl acetate-hexane). Unfortunately, no product was obtained at room temperature after 48 h (Table 1, entries 1). However, when the mixture reaction was heated at 70°C gratifyingly, we observed that the acceptable product was formed in an isolated yield of 84% within 5 h (Table 1, entry 2). Upon the construction of the desired outcome, immediately the precipitated solid was filtered off, washed with ethanol, and crystallized from hot ethanol in excellent yield. Amazingly, instead of the expected spiro pyridine product **11** (Scheme 4), we observed an unanticipated process leading to pyrrolo[3,4-c]quinolone **7a** in excellent yield (Scheme 2). On the other hand, post-workup product **7a**, the solvent was evaporated. We observed the precipitated white solid of pyrazole (from hydrazine and ethyl acetoacetate) slowly settling in which it was filtered and washed with water. The absolute structure of the newly synthesized product **7a** was explicitly confirmed by X-ray analysis.

**TABLE 1:** Examining; optimum reaction conditions.

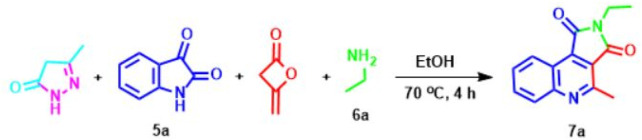				
Entry	Solvent	Temp. (˚C)	Time (h)	Yield (%)^b^
1	Ethanol (4 mL)	r.t.	48	-
2	Ethanol (4 mL)	70	4	90
3	Ethanol (4 mL)	80	4	90
4	Acetonitrile (4 mL)	70	12	45
5	Methanol (4 mL)	70	8	33
6	Water (4 mL)	70	8	52
7	Water/ethanol (4 mL)	70	5	60
8	Tetrahydrofuran(4 mL)	70	12	30

Then, in a controlled testing, the reaction of this five-component manufacturing process proceeds in the absence of pyrazole resulting from hydrazine and ethyl acetoacetate in which the effect of pyrazole was evaluated for this reaction. We concluded that the response could not advance without pyrazole under these conditions, and when the reaction mixture was carried with pyrazole (1 mol), the objective compound **7a** obtained an 84% yield. Also, the change in amounts of pyrazole was explored for the reaction. The best result (90%) of the product was formed when (1 mol) of the pyrazole was exploited. By decreasing the amount of pyrazole to (0.7 and 0.5 mol), the development was accomplished at 35% and 27%, and it was observed that increasing the pyrazole loading had a considerable effect on the formation product. However, without using pyrazole, the reaction failed to develop even after 48 h.

Thus, to increase the yield of pyrrolo[3,4-c]quinoline-1,3-dione **7a** and minimize reaction time, four-component reactions between isatin **5a**, pyrazole, diketene, and ethylamine **6a** were designed (Table 1), because of the success achieved using pyrazole promoted response. We found pyrrolo[3,4-c]quinoline-1,3-dione 7a as the only product when the reaction mixture was composed of a 1:1:1:1 variety of compounds.

In this reaction, solvent and temperature were examined to optimize the reaction conditions. Due; to the impossibility of carrying out the reaction at ambient temperature, the response was performed under reflux conditions. Also, it was observed that increasing the temperature above 70°C has no significant effect on the product yield. Despite obtaining good results, organic solvents did not improve much compared to water, including acetonitrile, methanol, water, water/ethanol, and tetrahydrofuran). Therefore, all reactions were performed under reflux conditions at 70°C in water to give satisfactory and excellent results.

Reaction conditions: pyrazole (1.0 mmol), isatine **5a** (1.0 mmol), diketene (1.0 mmol), ethyl amine **6a** (1.0 mmol), solvent (4.0 ml). [b] Isolated yield.

**SCHEME 3:**
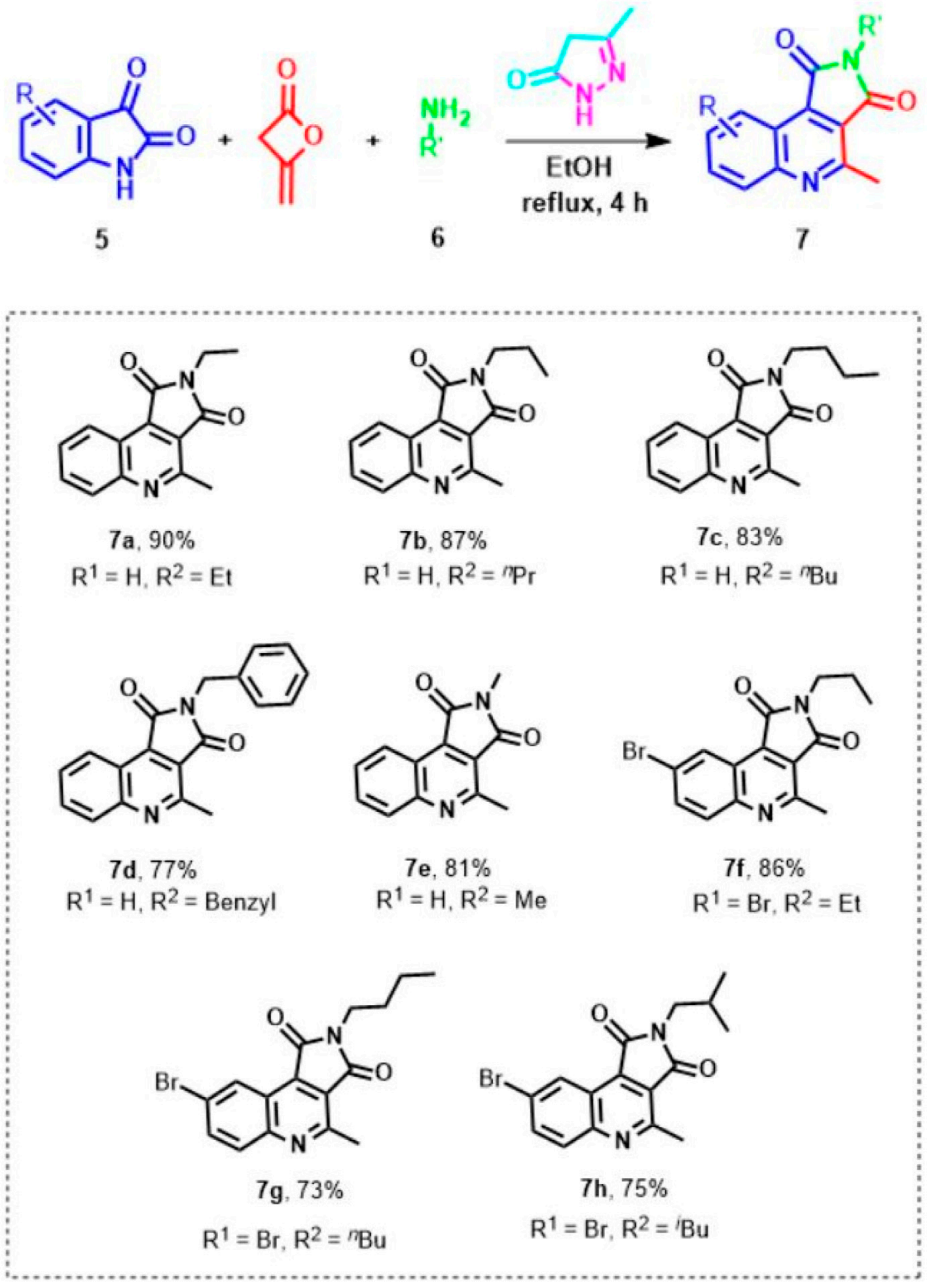
Scope; of the reaction.^a,b^.^a^[a] The reaction was performed at 70°C with 1 equivalent of substrates. [b] The new pyrrolo[3,4-c]quinoline-1,3-diones were afforded (see the Supporting Information).

Having identified the best available conditions, to explore the efficiency and generality of this approach, the reactions between another isatine **1** and primary amines **2** were conducted, and the outcomes are shown in Scheme 3. The corresponding functionalized pyrrolo[3,4-c]quinoline-1,3-diones **7** were obtained in excellent yields at 70°C in ethanol in the presence of pyrazole (1 mmol) as a promoter. Various primary amines (**6a-f**) reacted with isatines to generate corresponding pyrrolo[3,4-c]quinoline-1,3-diones **7a-h**.

**FIGURE 2:**
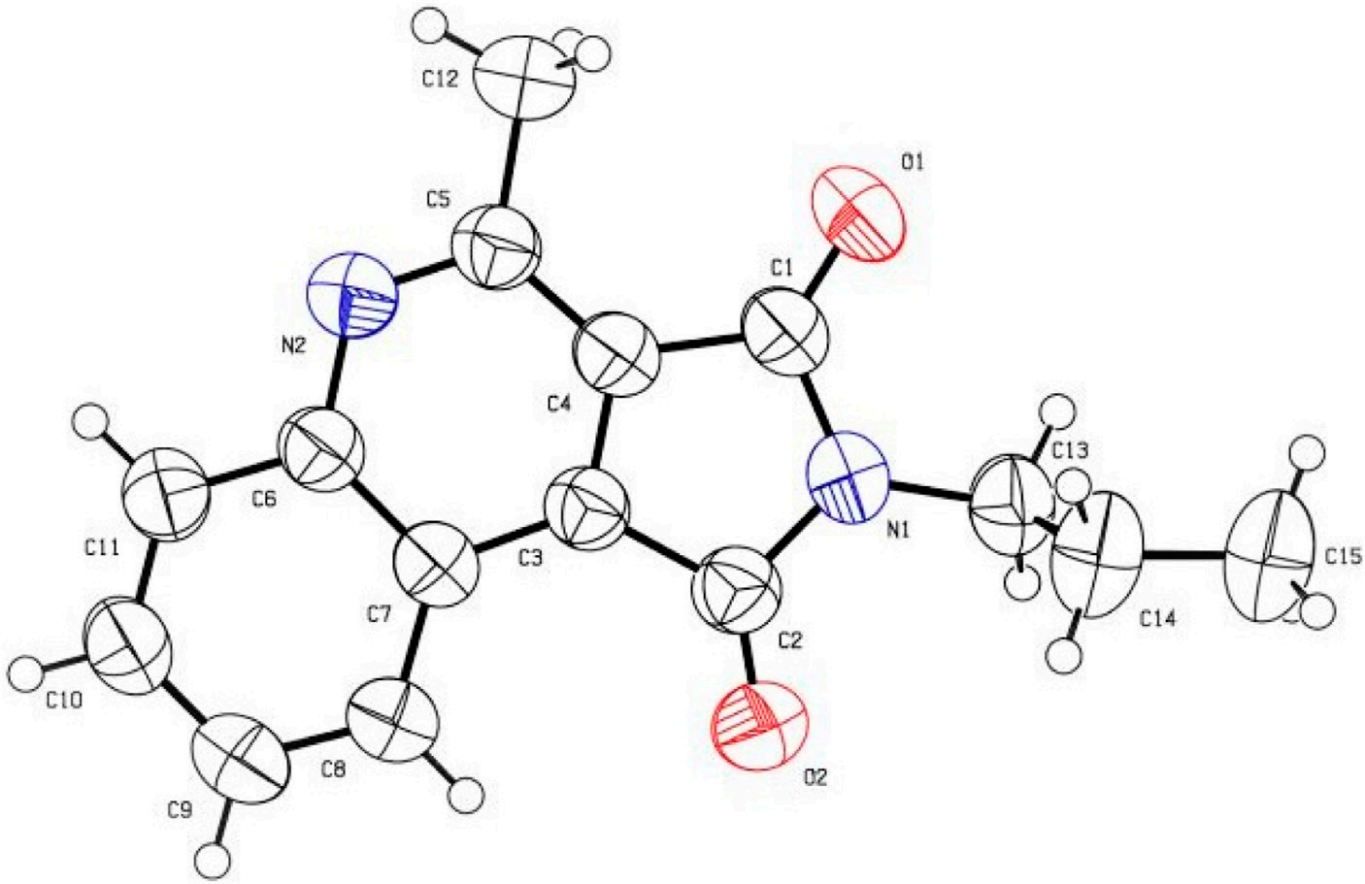
ORTEP; diagram of **7b**.

The skeleton of all synthetic compounds **7a-h** was elucidated by ESI-MS, IR, ^1^HNMR, ^13^CNMR spectroscopy, and X-ray analysis. FTIR of **7a** exhibited absorption bands in 1764, 1705, and 1622 due to the two CO and C=N stretching frequencies. In the ^1^H-NMR spectrum of **7a**, triplet and quartet in *δ* = 1.31 (^3^ *J*_H-H_ = 7.2 Hz) and *δ* = 3.77 (^3^ *J*_H-H_ = 7.2 Hz) ppm are due to CH_3_ and CH_2_ groups. The Singlet peak in *δ* = 3.01 is due to the CH_3_ group. Also the signals in the aromatic section confirmed the presence of the four aromatic hydrogens of the aromatic ring. The presence of 14 apparent signs in the ^13^C-NMR spectrum is in accordance with the suggested structure of **7a**. The highlighted areas in the ^13^C NMR are due to two CH_3_, two CH_2_, and two C=O groups, which are evident at *δ* = 13.94, 22.03, 33.01, 168.06, 168.33 ppm. Single crystal X-ray crystallography structure of **7b** was certified as the product structure (Figure 2).

**SCHEME 4:**
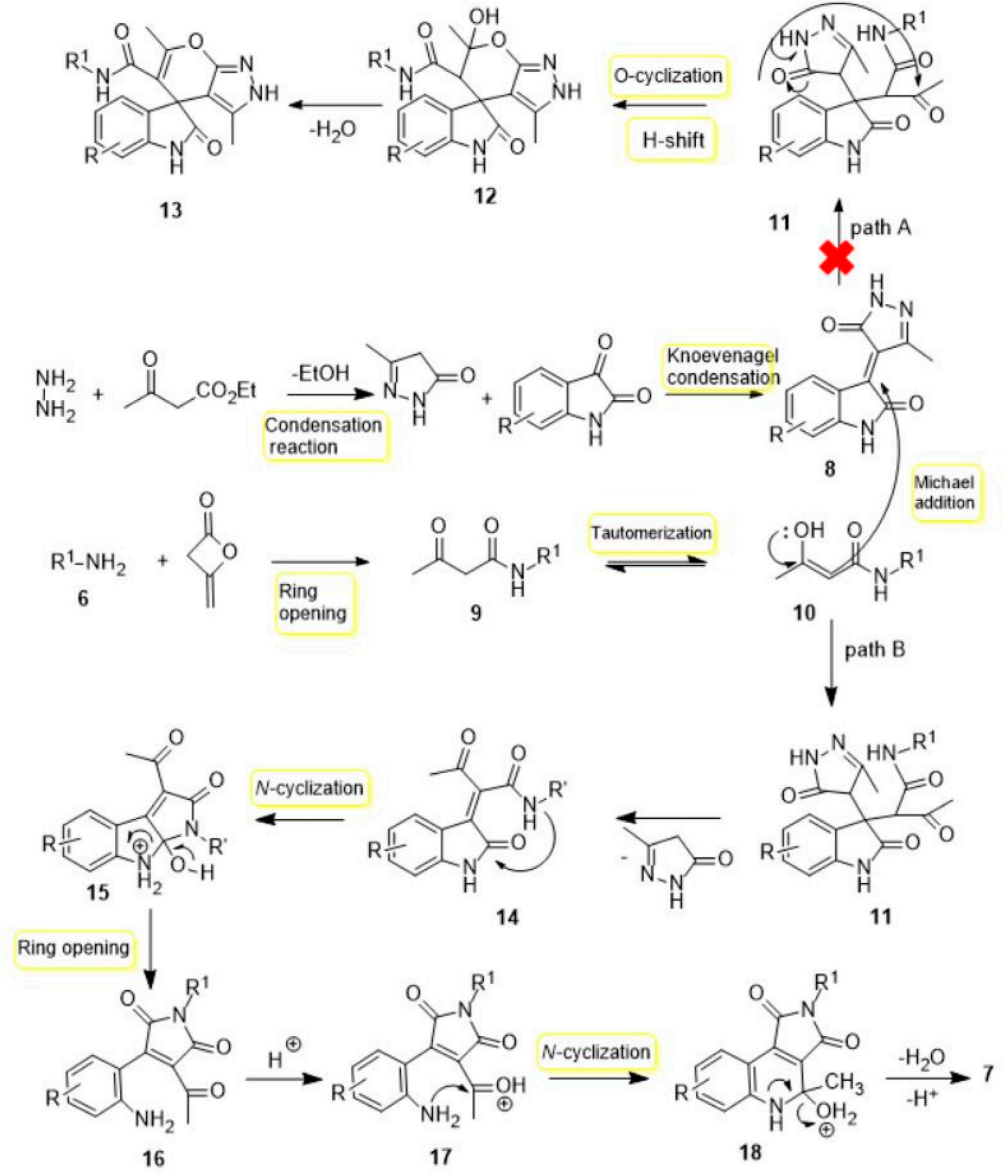
A plausible mechanism for the formation of the product **7**.

This reaction is a particular case, and a probable tool is illustrated in Scheme 4 for the generation of compound **7**. It is advisable to suggest that the first stage starts via a Knoevenagel-type condensation of isatine and pyrazole to provide the intermediate **8** as the Michael acceptor. Then, nucleophilic attack of the amine to diketene and ring-opening of diketene pursue by proton transfer to give *β*-ketoamide **9.** After the formation of adduct **8**, nucleophilic addition enol form **10** on the Michael acceptor **8** afforded intermediate **11** *via* Michael addition. At this stage, attending to the preceding articles, we expected adduct **11** with *O*-cyclization, attacking the carbonyl group, and tautomerization to give the desired heterocyclic compound **13**. But contemplating X-ray diffraction and ^1^H and ^13^C NMR spectra, product **12** was not formed, and another extraordinary incident happened. In truth, in this step, nucleophilic addition of the enol form **10** is followed the elimination of pyrazole to give **14** via proton transfer. Then, in intermediate **14**, with an intramolecular cyclization via *N*-nucleophilic attack of the amide group and proton transfer to form middle **15**, ring opening and proton transfer gives the medium **17**. Finally, nucleophilic attack of the amine **17** to C=O bond, intramolecular cyclization to form middle **18,** elimination of H_2_O, and deprotonation product **7** are created.

## 4 Conclusion

As a result, we have described an unusual three-component reaction to construct novel molecules containing a pyrrolo[3,4-c]quinoline-1,3-dione core from readily available reagents of pyrazole, isatine, diketene, and primary amine in ethanol. The most significant aspects of the process are the accessibility of the starting precursors, mild reaction conditions, short reaction times, high yields of the synthesized products, and easy operation at the manufacturing scale. The overall process of reaction includes all the aspects of green chemistry and has new portals for the growth of more sustainable multicomponent reactions. This category of heterocycles with several pharmacophores may be interesting for medicine and pharmacology.

## Data Availability

Detailed experimental procedures and compound characterization data in the Supporting Information (PDF) are available. X-ray Crystallography: Deposition Number 7a is 2202802c and contains the supplementary crystallographic data for this paper. www.ccdc.cam.ac.uk/structures.
