# Supplementary material for: Pyrazole‐promoted synthesis of pyrrolo[3,4‐c] quinoline-1,3‐diones in a novel diketene-based reaction
Source: Front Chem. 2023 Sep 26;11:1219986. doi: 10.3389/fchem.2023.1219986 (PMC10562593; doi:10.3389/fchem.2023.1219986)

**Supporting Information**

**The Table of Contents**

| Characteristic data for compounds **7a-h** | S1-S4 |
| --- | --- |
| Molecular structure of compound **7b** | S5-S8 |
| ^1^H, ^13^C NMR, IR, and Mass spectra of **7a-h** | S8-S32 |

**Characteristic data for compounds 7a-h**


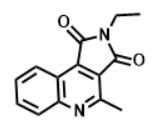


**2-ethyl-4-methyl-1H-pyrrolo[3,4-c]quinoline-1,3(2H)-dione (7a).** Light yellow, mp= 138-140 ℃. 0.216 g, Yield: 90%. Anal. calcd. For C_14_H_12_N_2_O_2_ (240): C, 69.99; H, 5.03; N, 11.66. Found: C, 69.70; H, 5.01; N, 11.67. IR (KBr) (v_max_, cm^-1^): 1622 (C=N) 1705 (C=O) 1764 (C=O). ^1^H NMR (300 MHz, CDCl_3_-d): *δ* = 1.31 (t, *^3^J* = 7.2 Hz, 3H, CH_3_), 3.01 (s, 3H, CH_3_), 3.77 (q, *^3^J* = 7.2 Hz, 2H, CH_2_), 7.66 (ddd, *^3^J* = 8.2, 6.9, 1.2 Hz, 1H, CH of Ar), 7.83 (ddd, *^3^J* = 8.6, 6.9, 1.5 Hz, 1H, CH of Ar), 8.07 (dt, *^3^J* = 8.6, 1.0 Hz, 1H, CH of Ar), 8.74 (ddd, *^3^J* = 8.3, 1.5, 0.7 Hz, 1H, CH of Ar). ^13^C NMR (75 MHz, CDCl_3_-d): δ = 13.94 (CH_3_), 22.03 (CH_3_), 33.01 (CH_2_), 120.53 (CH of Ar), 122.02 (C*_ipso_* of Ar), 124.78 (CH of Ar), 128.75 (CH of Ar), 129.16 (CH of Ar), 132.47 (*C*-C=O), 136.05 (*C*-C=O), 151.41 (C*_ipso_* of Ar), 154.82(C=N), 168.06 (C=O), 168.33 (C=O).

**
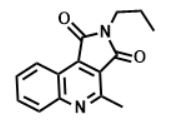
**

**4-methyl-2-propyl-1H-pyrrolo[3,4-c]quinoline-1,3(2H)-dione (7b).** Light yellow, mp= 99-101 ℃. 0.22 g, Yield: 87%. Anal. calcd. for C_15_H_14_N_2_O_2_ (254): C, 70.85; H, 5.55; N, 11.02 %. Found: C, 70.86; H, 5.54; N, 11.02 %. IR (KBr) (v_max_, cm^-1^): 1763 (C=O) 1712 (C=O) 1623 (C=N). ^1^H NMR (500 MHz, CDCl_3_-d): *δ* = 0.99 (t, *^3^J* = 7.4 Hz, 3H, CH_3_), 1.74 (td, 2H *, ^3^J* = 14.8, 7.4 Hz, CH_2_), 3.04 (s, 3H, CH_3_), 3.70 (t, 2H, *^3^J* = 7.3 Hz, , CH_2_), 7.69 (t, *^3^J* = 7.6 Hz, 1H, CH of Ar), 7.81 – 7.90 (m, 1H, CH of Ar), 8.11 (d, *^3^J* = 8.6, 1H, CH of Ar), 8.79 (d, ^3^*J* = 8.3 Hz, 1H, CH of Ar). ^13^C NMR (126 MHz, CDCl_3_-d): δ = 11.35 (CH_3_), 21.93 (CH_2_), 22.08 (CH_3_), 39.74 (CH_2_), 120.63 (CH of Ar), 121.99 (C*_ipso_* of Ar), 124.88 (CH of Ar), 128.81 (CH of Ar), 129.22 (CH of Ar), 132.55 (*C*-C=O), 136.05 (*C*-C=O), 151.53 (C*_ipso_* of Ar), 152.92 (C=N), 168.40 (C=O), 168.63 (C=O).


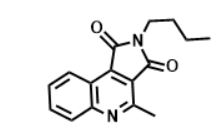


**2-butyl-4-methyl-1H-pyrrolo[3,4-c]quinoline-1,3(2H)-dione (7c).** Light yellow, mp= 84-86 ℃. 0.22 g, Yield: 83%. Anal. calcd. For C_16_H_16_N_2_O_2_ (268): C, 71.62; H, 6.01; N, 10.44. Found: C, 71.61; H, 6.03; N, 10.43. IR (KBr) (v_max_, cm^-1^): 1623 (C=N) 1715 (C=O) 1763 (C=O). ^1^H NMR (500 MHz, CDCl_3_-d): *δ* = 0.97 (t, *^3^J* = 7.3, 3H, CH_3_), 1.36 – 1.44 (m, 2H, CH_2_), 1.68- 1.73 (m, 2H, CH_2_), 3.04 (s, 3H, CH_3_), 3.73 (t, *^3^J* = 7.3, 2H, CH_2_), 7.65– 7.73 (m, 1H, CH of Ar), 7.80 – 7.90 (m, 1H, CH of Ar), 8.11 (d, *^3^J* = 8.6, 1H, CH of Ar), 8.79 (d, ^3^*J* = 8.4 Hz, 1H, CH of Ar). ^13^C NMR (126 MHz, CDCl_3_-d): δ = 13.62 (CH_3_), 20.09 (CH_2_), 22.07 (CH_3_), 30.66 (CH_2_), 37.91 (CH_2_), 120.63 (CH of Ar), 122.01 (C*_ipso_* of Ar), 124.88 (CH of Ar), 128.81 (CH of Ar), 129.22 (CH of Ar), 132.53 (*C*-C=O), 136.07 (*C*-C=O), 151.52 (C*_ipso_* of Ar), 154.90 (C=N), 168.37 (C=O), 168.62 (C=O). MS (EI, 70 eV): *m/z* (%) = 268.1 (83.2), 169.1 (28), 140.1 (28), 197.1 (6.4), 115.1 (10.4).

**
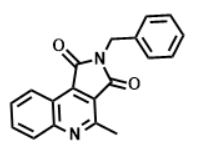
**

**2-benzyl-4-methyl-1H-pyrrolo[3,4-c]quinoline-1,3(2H)-dione (7d).** Light yellow, mp= 180-182 ℃. 0.234 g, Yield: 77%. Anal. calcd. for C_19_H_14_N_2_O_2_ (302): C, 75.48; H, 4.67; N, 9.27. Found: C, 75.49; H, 4.68; N, 9.25. IR (KBr) (v_max_, cm^-1^): 1625 (C=N) 1712 (C=O) 1768(C=O). ^1^H NMR (300 MHz, CDCl_3_-d): *δ* = 2.98 (s, 3H, CH_3_), 4.83 (s, 2H, CH_2_), 7.19 – 7.42 (m, 3H, 3CH of Ar), 7.42 – 7.51 (m, 2H, 2CH of Ar), 7.58 (ddd, *^3^J* = 8.2, 6.9, 1.2 Hz, 1H, CH of Ar), 7.74 (ddd, *^3^J* = 8.5, 6.9, 1.5 Hz, 1H, CH of Ar), 7.99 (d, *^3^J* = 8.6 Hz, 1H, CH of Ar), 8.66 (dd, *^3^J* = 8.4, 1.6 Hz, 1H, CH of Ar). ^13^C NMR (75 MHz, CDCl_3_-d): δ = 22.03 (CH_3_), 41.66 (CH_2_), 120.36 (CH of Ar), 121.79 (C*_ipso_* of Ar), 124.72 (CH of Ar), 127.98 (2CH of Ar), 128.73 (4CH of Ar), 129.12 (C*_ipso_* of Ar), 132.50 (CH of Ar), 135.75 (*C*-C=O), 136.05 (*C*-C=O), 151.31 (C*_ipso_* of Ar), 154.85 (C=N), 167.78 (C=O), 168.03 (C=O).


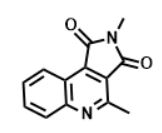


**2,4-dimethyl-1H-pyrrolo[3,4-c]quinoline-1,3(2H)-dione (7e).** Light yellow, mp= 153-155 ℃. 0.184 g, Yield: 81%. Anal. calcd. for C_19_H_14_N_2_O_2_ (226): C, 69.02; H, 4.46; N, 12.38. Found: C, 69.01; H, 4.49; N, 12.34. IR (KBr) (v_max_, cm^-1^): 1622 (C=N) 1707 (C=O) 1766 (C=O). ^1^H NMR (300 MHz, CDCl_3_-d): *δ* = 2.95 (s, 3H, CH_3_), 3.17 (s, 3H, CH_3_), 7.62 (ddd, *^3^J* = 8.2, 6.9, 1.2 Hz, 1H, CH of Ar), 7.80 (ddd, *^3^J* = 8.6, 6.9, 1.5 Hz, 1H, CH of Ar), 8.01 (dt, *^3^J* = 8.6, 1.0 Hz, 1H, CH of Ar), 8.65 (ddd, *^3^J* = 8.4, 1.5, 0.7 Hz, 1H, CH of Ar). ^13^C NMR (75 MHz, CDCl_3_-d): δ = 21.99 (CH_3_), 23.90 (CH_3_), 120.32 (CH of Ar), 121.93 (C*_ipso_* of Ar), 124.69 (CH of Ar), 128.74 (CH of Ar), 129.12 (CH of Ar), 131.46 (*C*-C=O), 135.90 (*C*-C=O), 151.33 (C*_ipso_* of Ar), 154.73 (C=N), 168.10 (C=O), 168.38 (C=O).


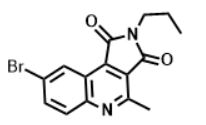


**8-bromo-4-methyl-2-propyl-1H-pyrrolo[3,4-c]quinoline-1,3(2H)-dione (7f).** Light yellow, mp= 143-145℃. 0.287 g, Yield: 86%. Anal. calcd. for C_15_H_13_BrN_2_O_2_ (332): C, 54.07; H, 3.93; Br, 23.98; N, 8.41. Found: C, 54.06; H, 3.94; Br, 23.99; N, 8.40; IR (KBr) (v_max_, cm^-1^): 1619 (C=N) 1707 (C=O) 1763 (C=O). ^1^H NMR (300 MHz, CDCl_3_-d): *δ* = 0.97 (t, *^3^J* = 7.4 Hz, 3H, CH_3_), 1.69-1.79 (m, 2H, CH_2_), 2.98 (s, 3H, CH_3_), 3.68 (t, *^3^J* = 7.2 Hz, 2H, CH_2_), 7.81-7.98 (m, 2H, 2CH of Ar), 8.87 (d, ^3^J = 2.1 Hz, 1H, CH of Ar). ^13^C NMR (75 MHz, CDCl_3_-d): δ = 11.32 (CH_3_), 21.87 (CH_2_), 22.03 (CH_3_), 39.81 (CH_2_), 121.32 (CH of Ar), 122.50 (C*_ipso_* of Ar), 123.39 (CH of Ar), 126.88 (CH of Ar), 130.65 (*C*-C=O), 134.85 (C*_ipso_* of Ar), 135.97 (*C*-C=O), 149.84 (C*_ipso_* of Ar), 155.32 (C=N), 167.80 (C=O), 168.13 (C=O).


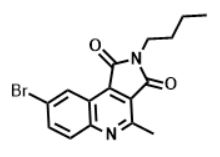


**8-bromo-2-butyl-4-methyl-1H-pyrrolo[3,4-c]quinoline-1,3(2H)-dione (7g).** Light yellow, mp= 131-133 ℃. 0.254 g, Yield: 73%. Anal. calcd. for C_16_H_15_BrN_2_O_2_ (346): C, 55.35; H, 4.35; Br, 23.01; N, 8.07. Found: C, 55.33; H, 4.36; Br, 23.01; N, 8.08. IR (KBr) (v_max_, cm^-1^): 1620 (C=N) 1712(C=O) 1768 (C=O). ^1^H NMR (300 MHz, CDCl_3_-d): *δ* = 0.93 (t, *^3^J* = 7.3 Hz, 3H, CH_3_), 1.29-1.41(m, 2H, CH_2_), 1.59-1.67 (m, 2H, CH_2_), 2.92 (s, 3H, CH_3_), 3.66 (t, *^3^J* = 7.3 Hz, 2H, CH_2_), 7.79 (Br.2H 2CH of Ar), 8.74 (Br, 1H, CH of Ar). ^13^C NMR (75 MHz, CDCl_3_-d): δ = 13.56 (CH_3_), 20.01 (CH_2_), 21.97 (CH_3_), 30.53 (CH_2_), 37.93 (CH_2_),121.12 (CH of Ar), 122.39 (C*_ipso_* of Ar), 123.31 (CH of Ar), 126.72 (CH of Ar), 130.55 (*C*-C=O), 134.68 (C*_ipso_* of Ar), 135.82 (*C*-C=O), 149.67 (C*_ipso_* of Ar), 155.22 (C=N), 167.63 (C=O), 167.91 (C=O).


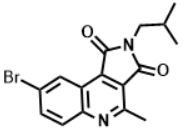


**8-bromo-2-isobutyl-4-methyl-1H-pyrrolo[3,4-c]quinoline-1,3(2H)-dione (7h).** Light yellow, mp= 118-120 ℃. 0.261 g, Yield: 75%. Anal. calcd. for C_16_H_15_BrN_2_O_2_ (346): C, 55.35; H, 4.35; Br, 23.01; N, 8.07. Found: C, 55.37; H, 4.34; Br, 23.01; N, 8.06. IR (KBr) (v_max_, cm^-1^): 1620 (C=N) 1713(C=O) 1767 (C=O). ^1^H NMR (300 MHz, CDCl_3_-d): *δ* = 0.97 (s, 3H, CH_3_), 0.99 (s, 3H, CH_3_), 2.14 (m, 1H, CH), 3.03 (s, 3H, CH_3_), 3.55 (d, *^3^J* = 7.3 Hz, 2H, CH_2_), 7.86 – 8.02 (m, 2H, 2 CH of Ar), 8.96 (d, *^3^J* = 2.1 Hz, 1H, CH of Ar). ^13^C NMR (75 MHz, CDCl_3_-d): δ = 20.06 (2CH_3_), 22.12 (CH_3_), 27.88 (CH), 45.53 (CH_2_), 121.44 (CH of Ar), 122.46 (C*_ipso_* of Ar), 123.43 (CH of Ar), 127.00 (CH of Ar), 130.71 (*C*-C=O), 134.85 (C*_ipso_* of Ar), 136.05 (*C*-C=O), 149.98 (C*_ipso_* of Ar), 155.38 (C=N), 168.07 (C=O), 168.38 (C=O).

**X-ray crystal structure determination of 7b**

Deposition Number for **7b** is 2202802c contains the supplementary crystallographic data for this paper. These data are provided free of charge by the joint Cambridge Crystallographic Data. Centre and Fachinformationszentrum Karlsruhe Access Structures service. [www.ccdc.cam.ac.uk/structures](http://www.ccdc.cam.ac.uk/structures).

**Preparation of single crystal**: We Prepared a solution of the pure compound **7b** in hot ethanol cool hexane (saturated or nearly saturated), and covered the container, but not tightly. We Gently put the container in a quiet, out of the way place and allowed the solvent to evaporate slowly. After five days, the yellow crystals formed.

***
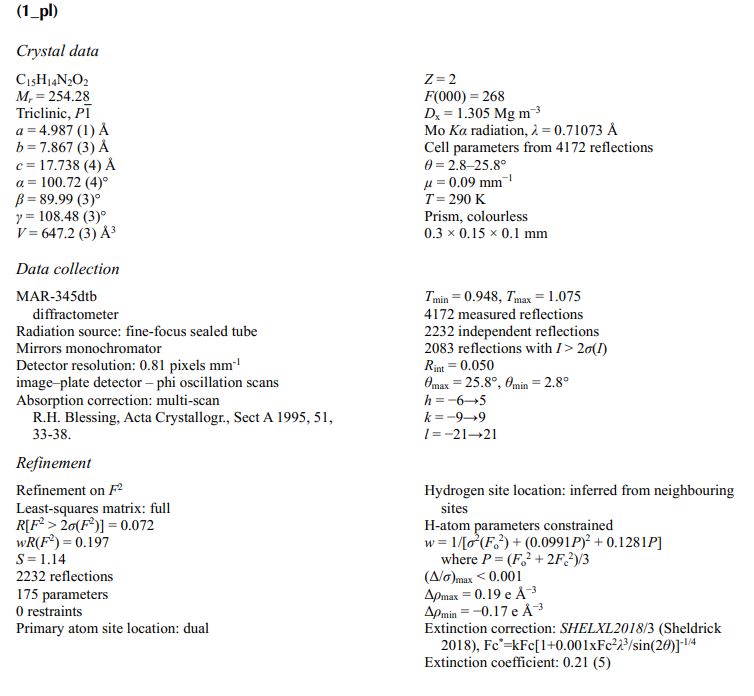
***

***
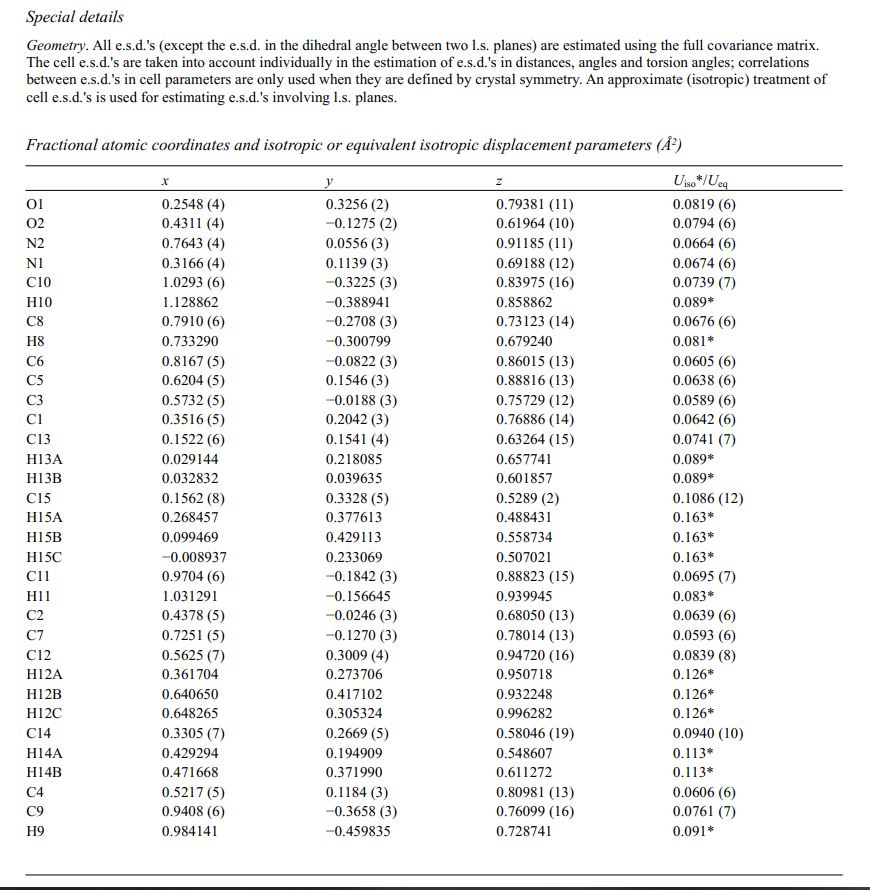
***

***
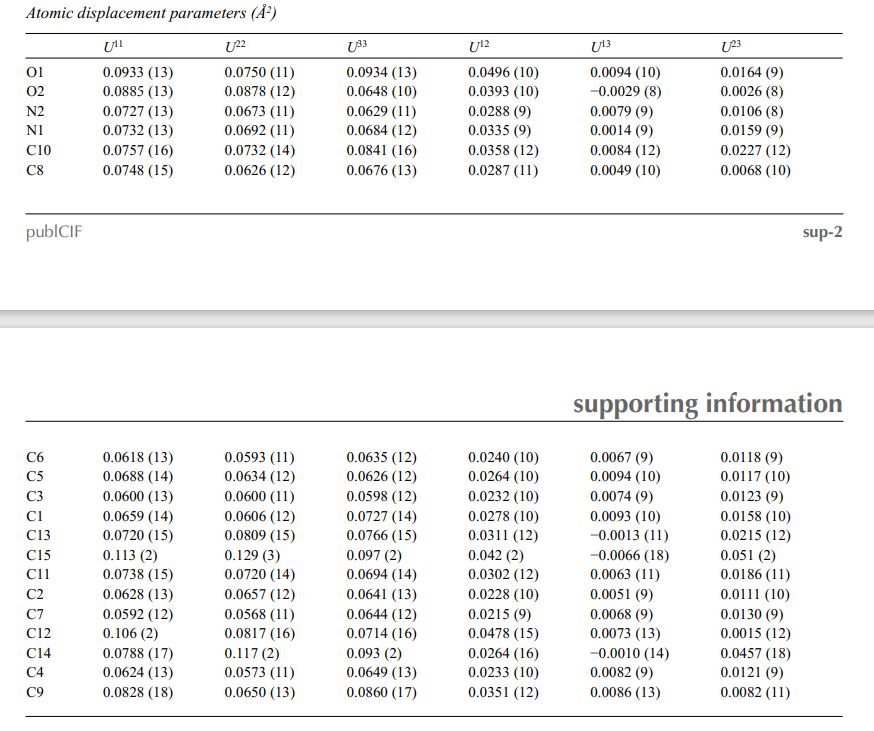
***

***
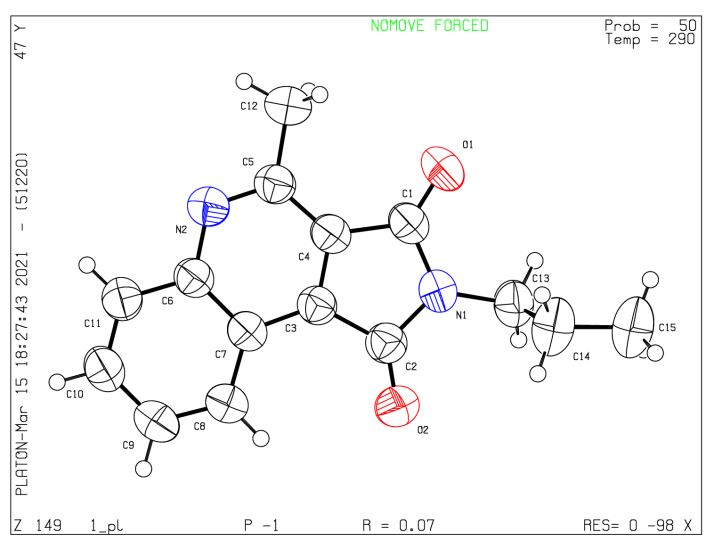
***

^1^H NMR of **7a**


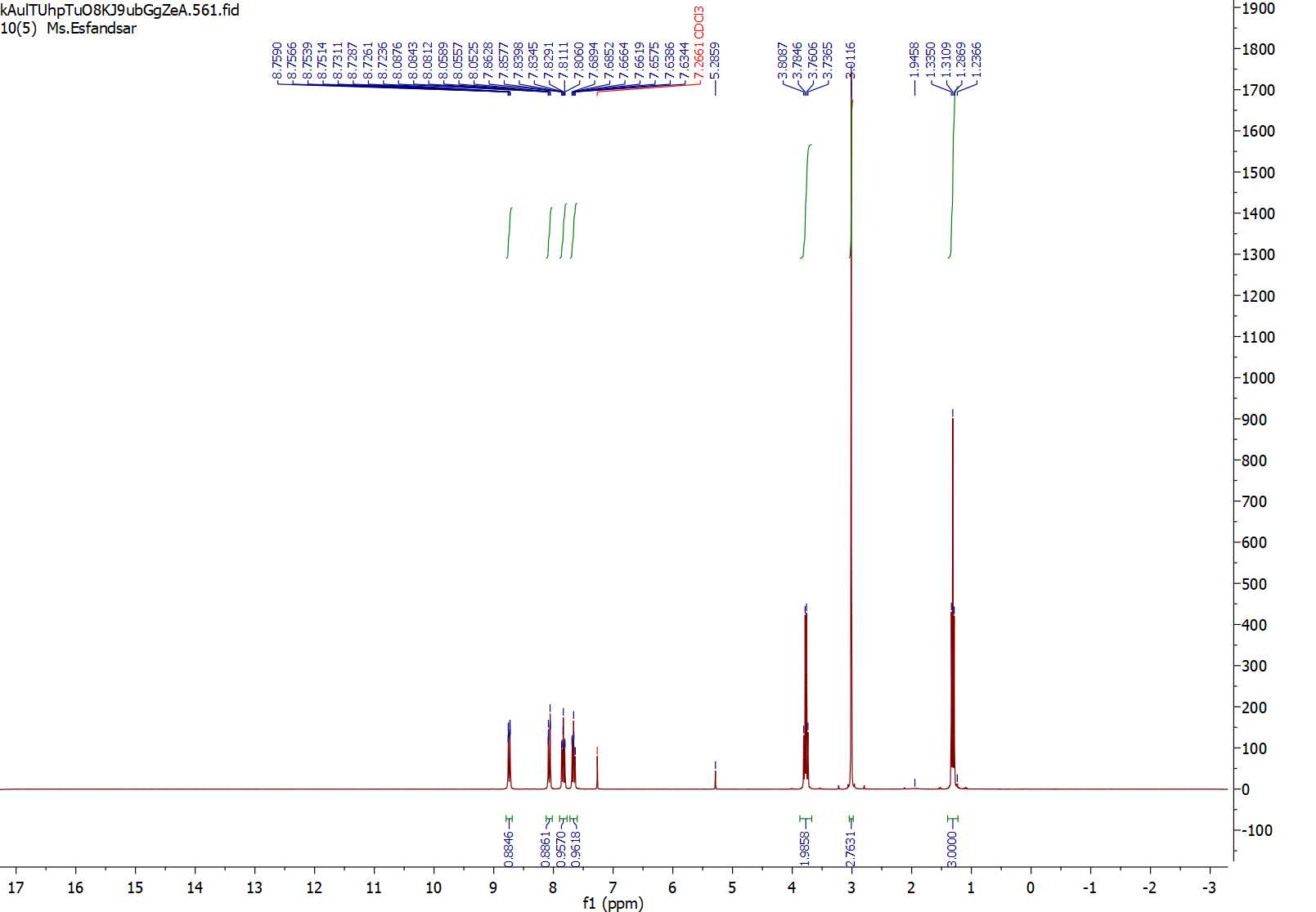


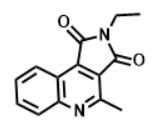


^13^C NMR of **7a**


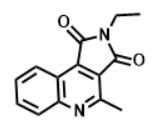

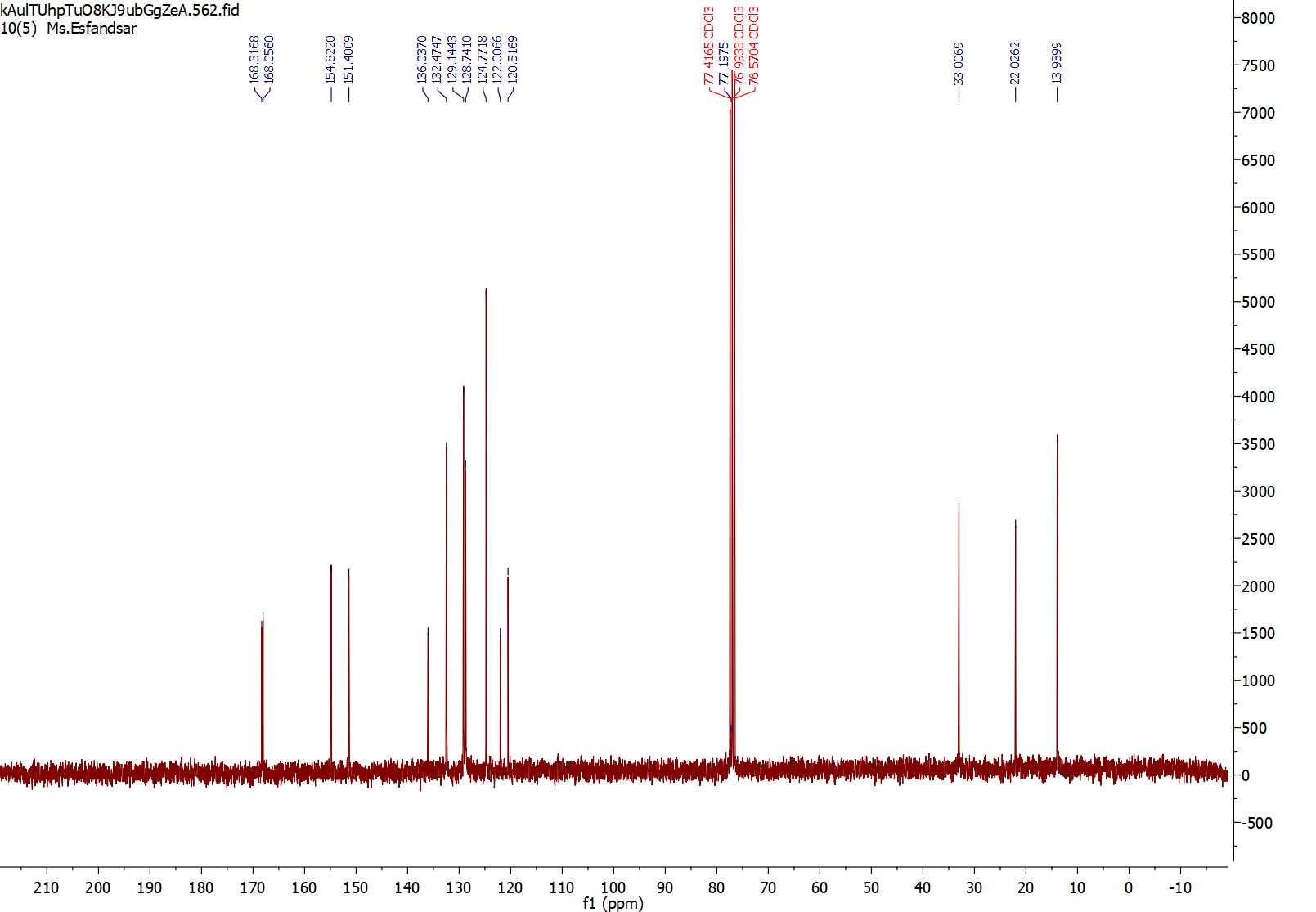


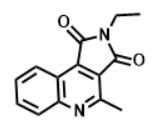
IR spectrum of **7a**

^1^H NMR of **7b**


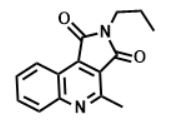

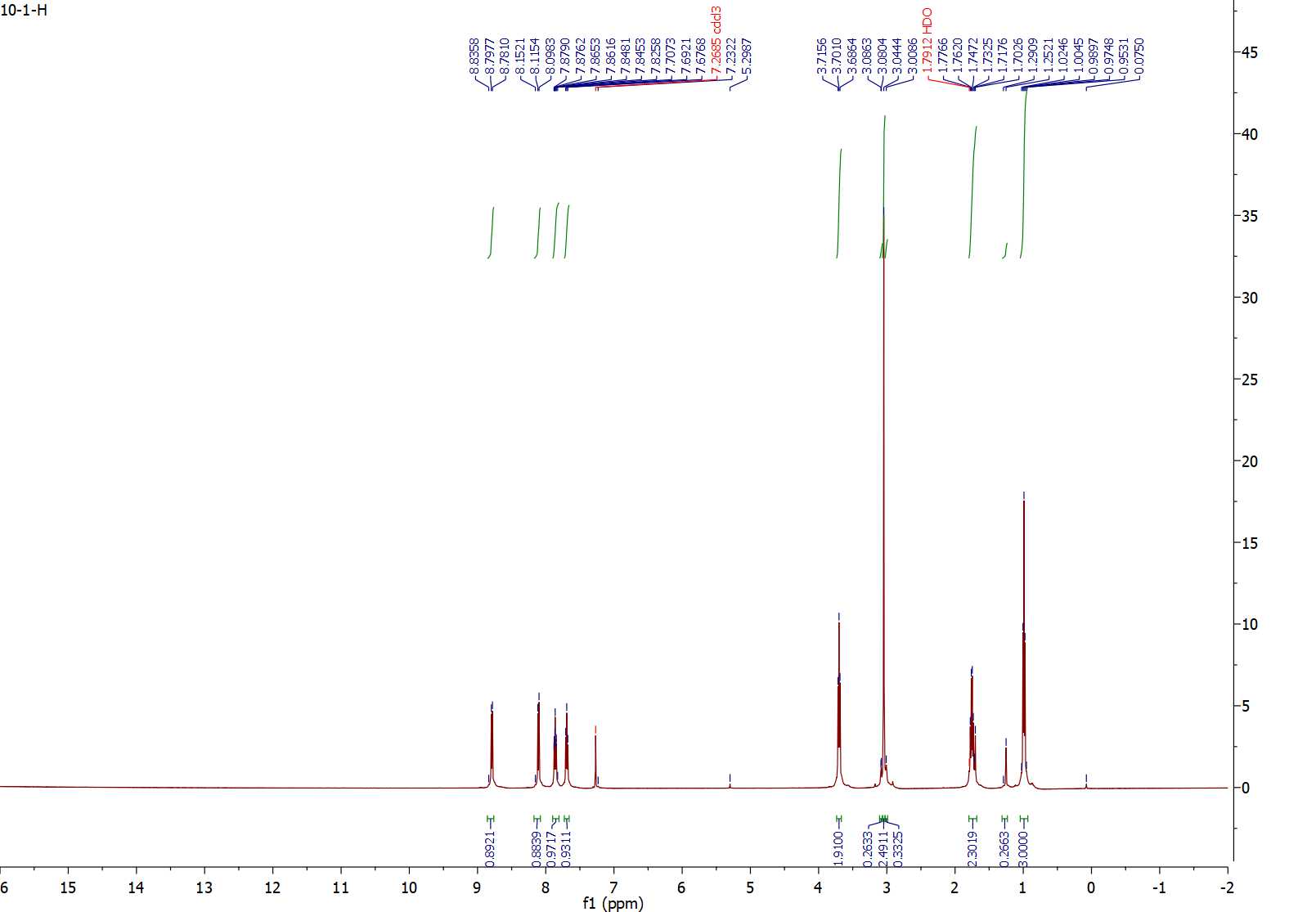
^13^C NMR of **7b**


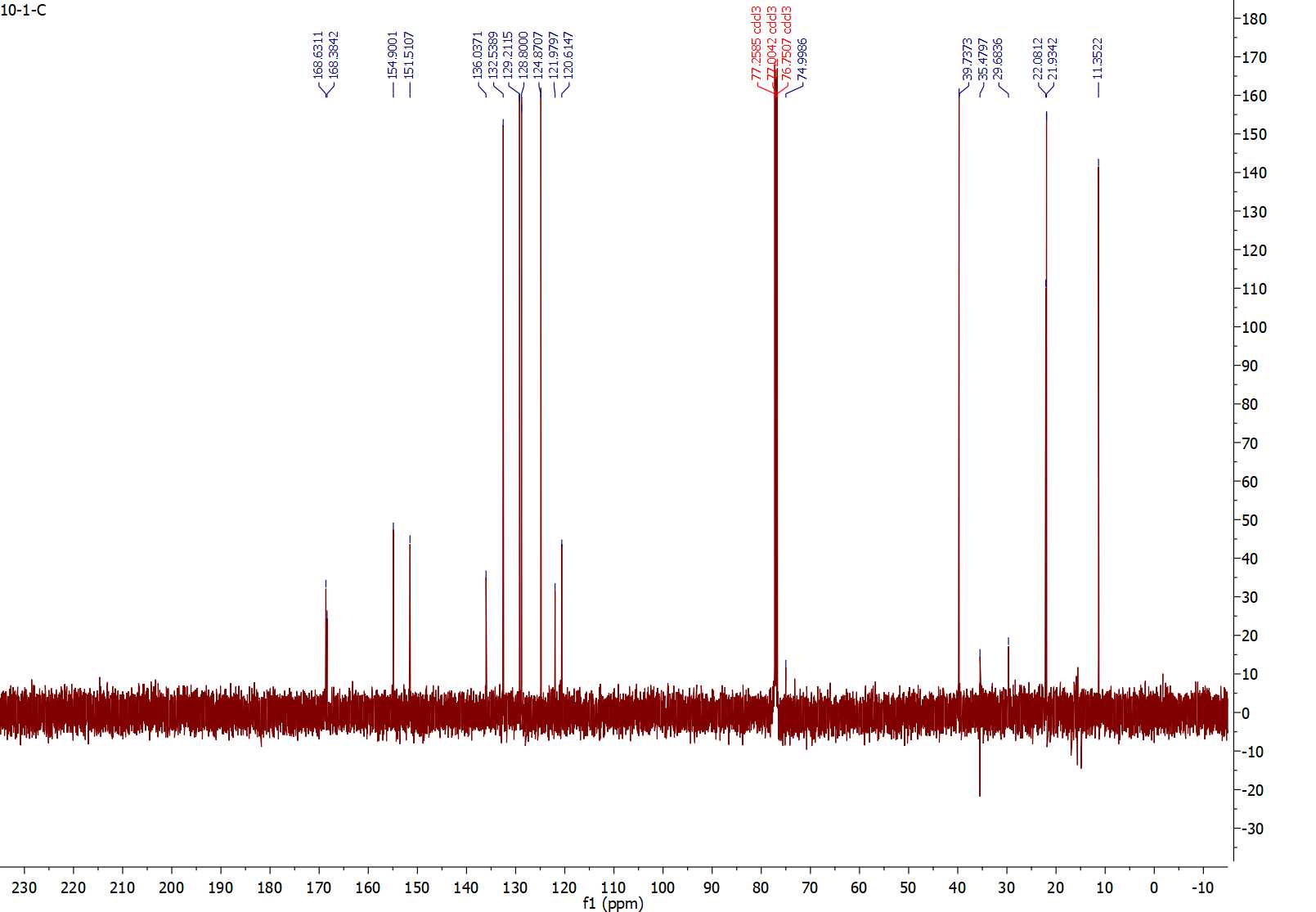


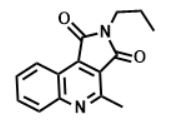


IR spectrum of **7b**

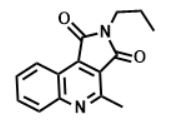


^1^H NMR of **7c**


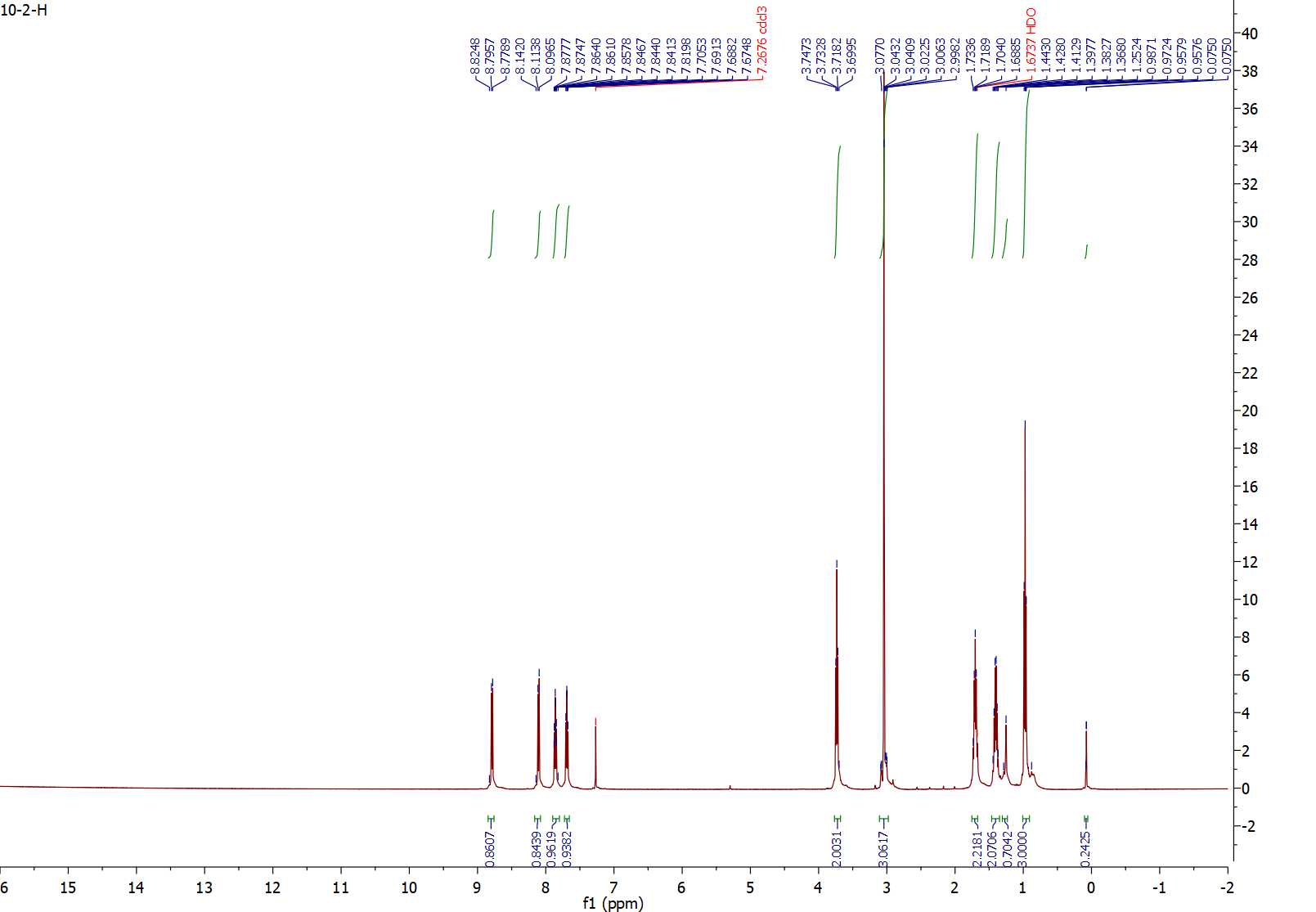


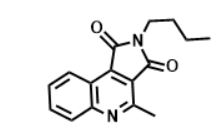


^13^C NMR of **7c**


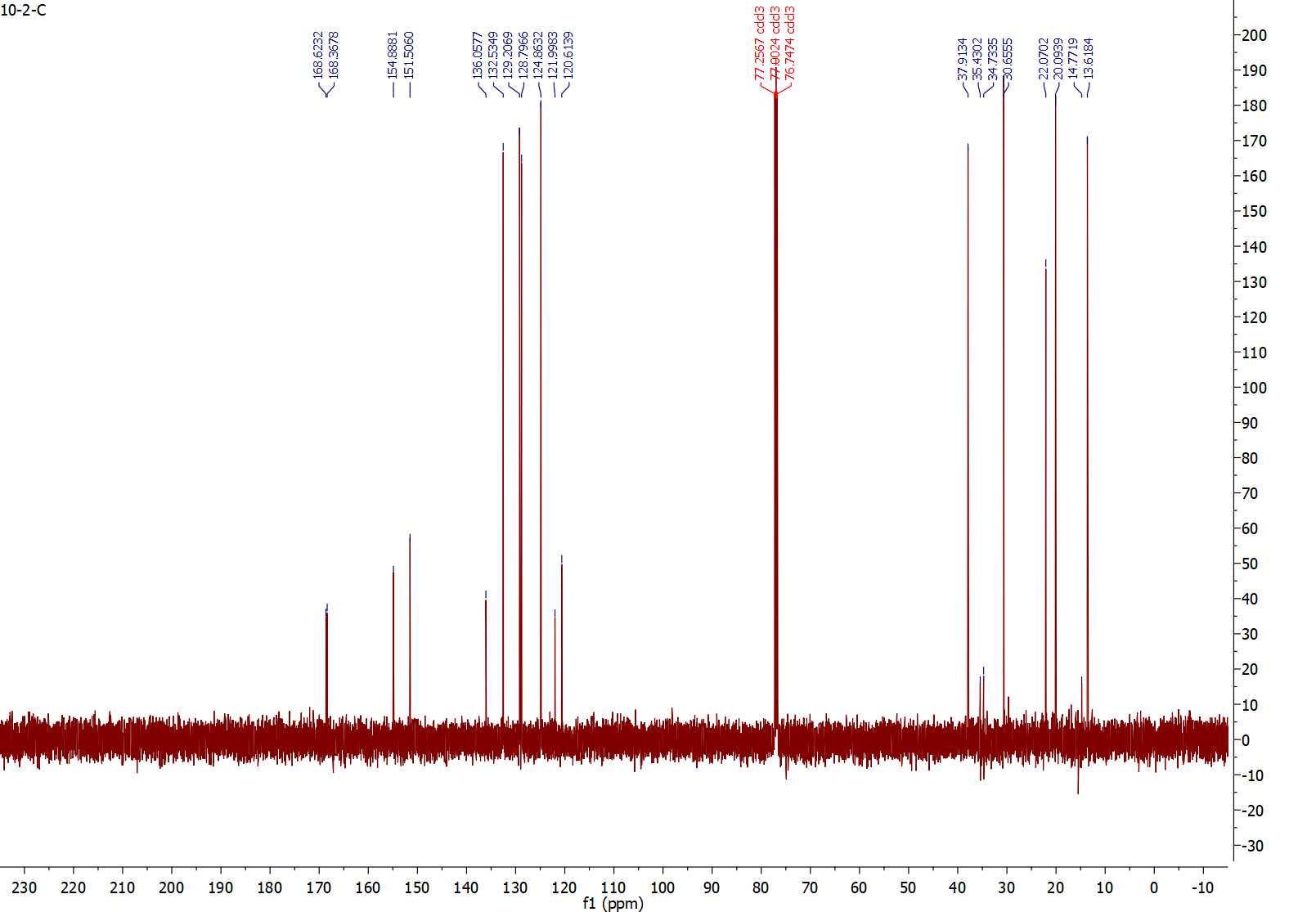


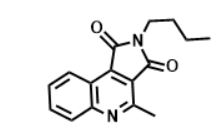


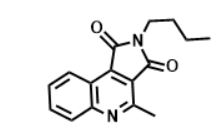
Mass spectrum of **
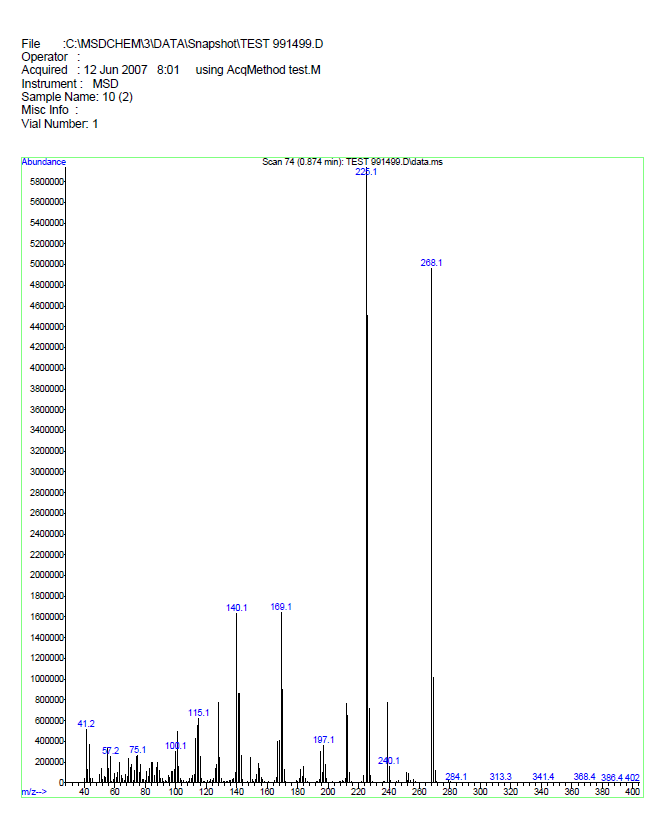
7c**


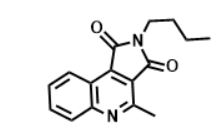
IR spectrum of **7c**


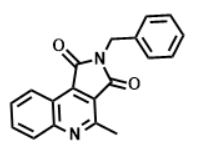

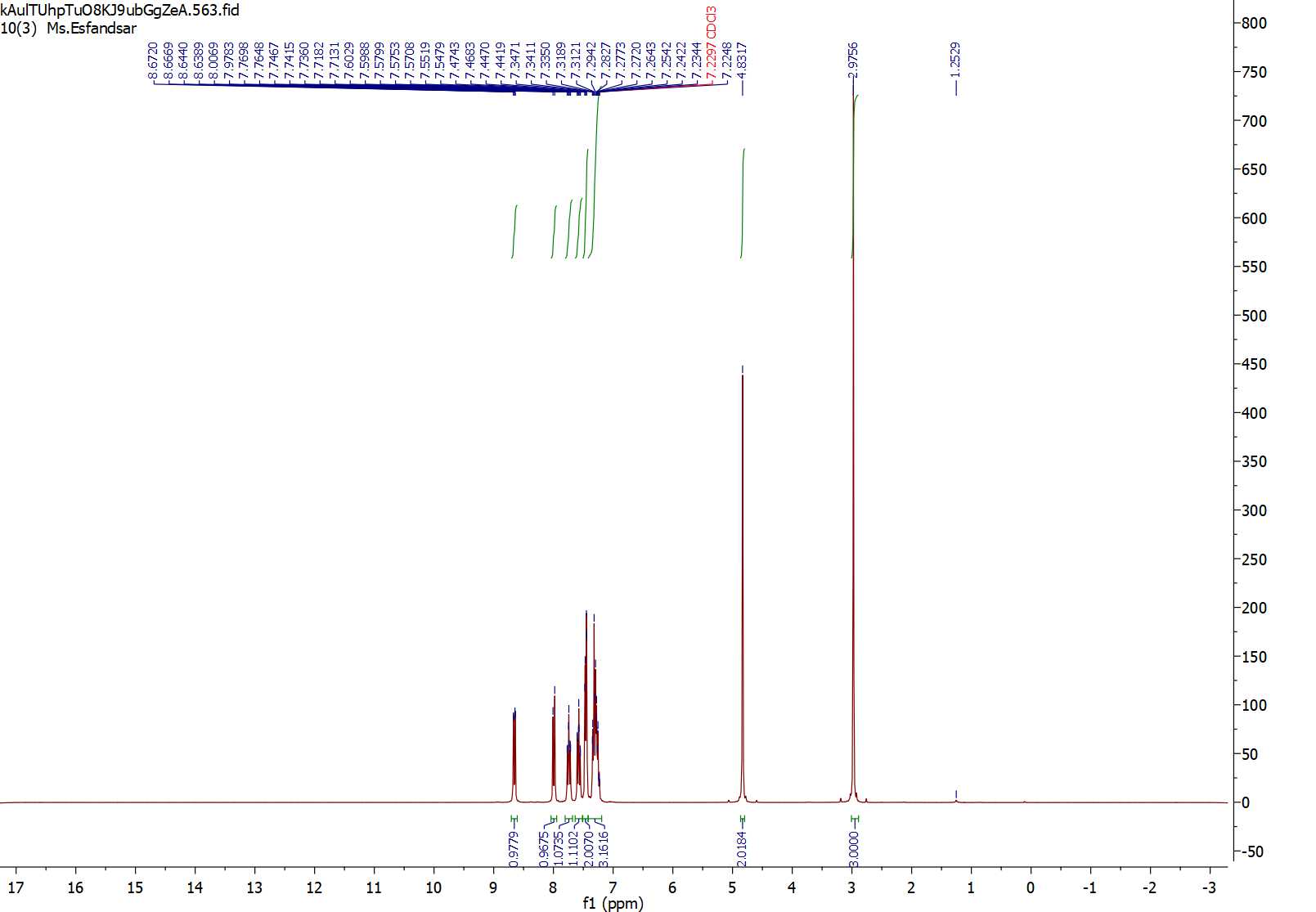
^1^H NMR of **7d**


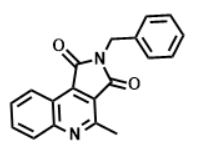

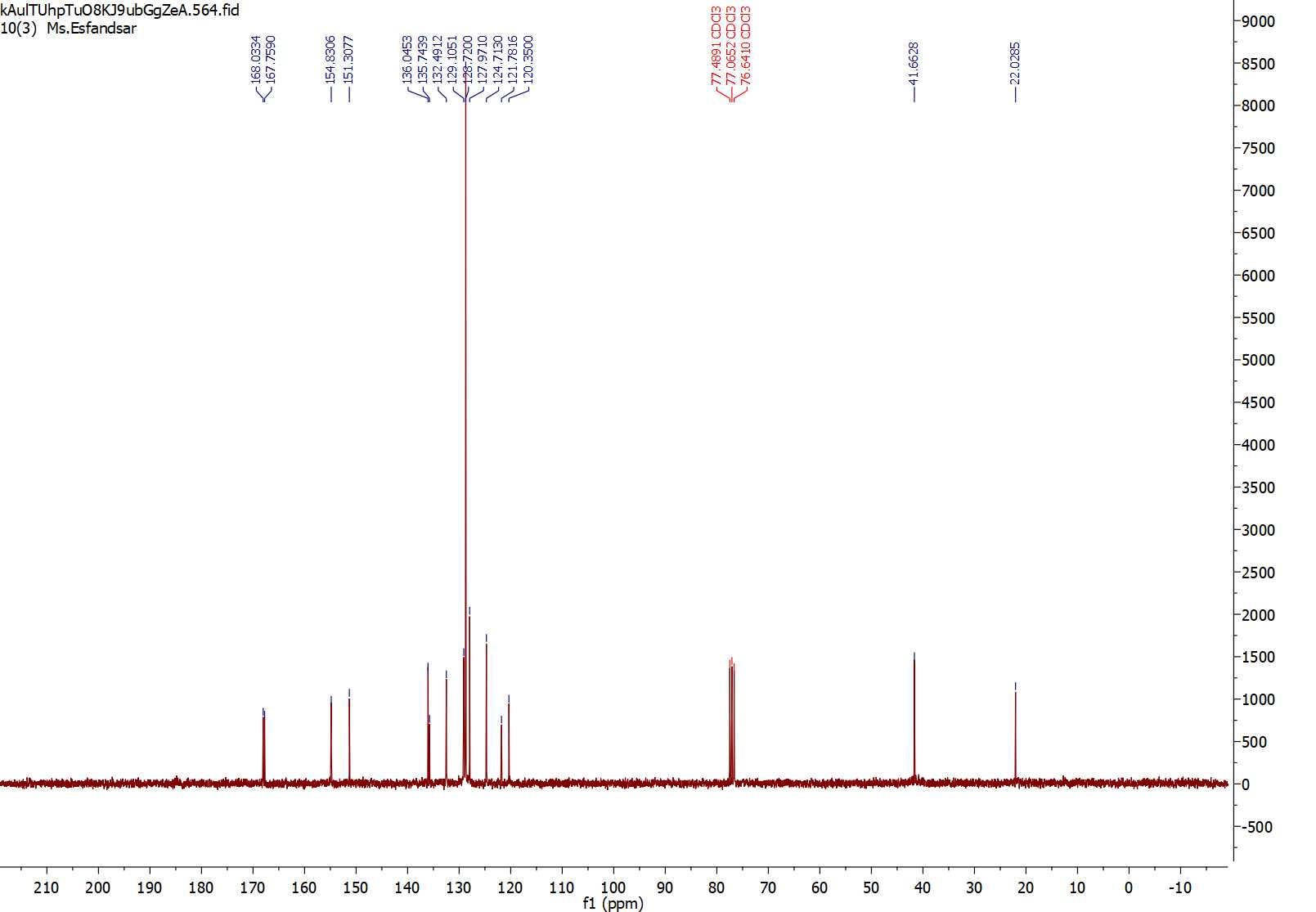
^13^C NMR of **7d**

IR spectrum of **7d**

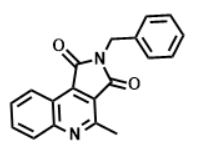


^1^H NMR of **7e**


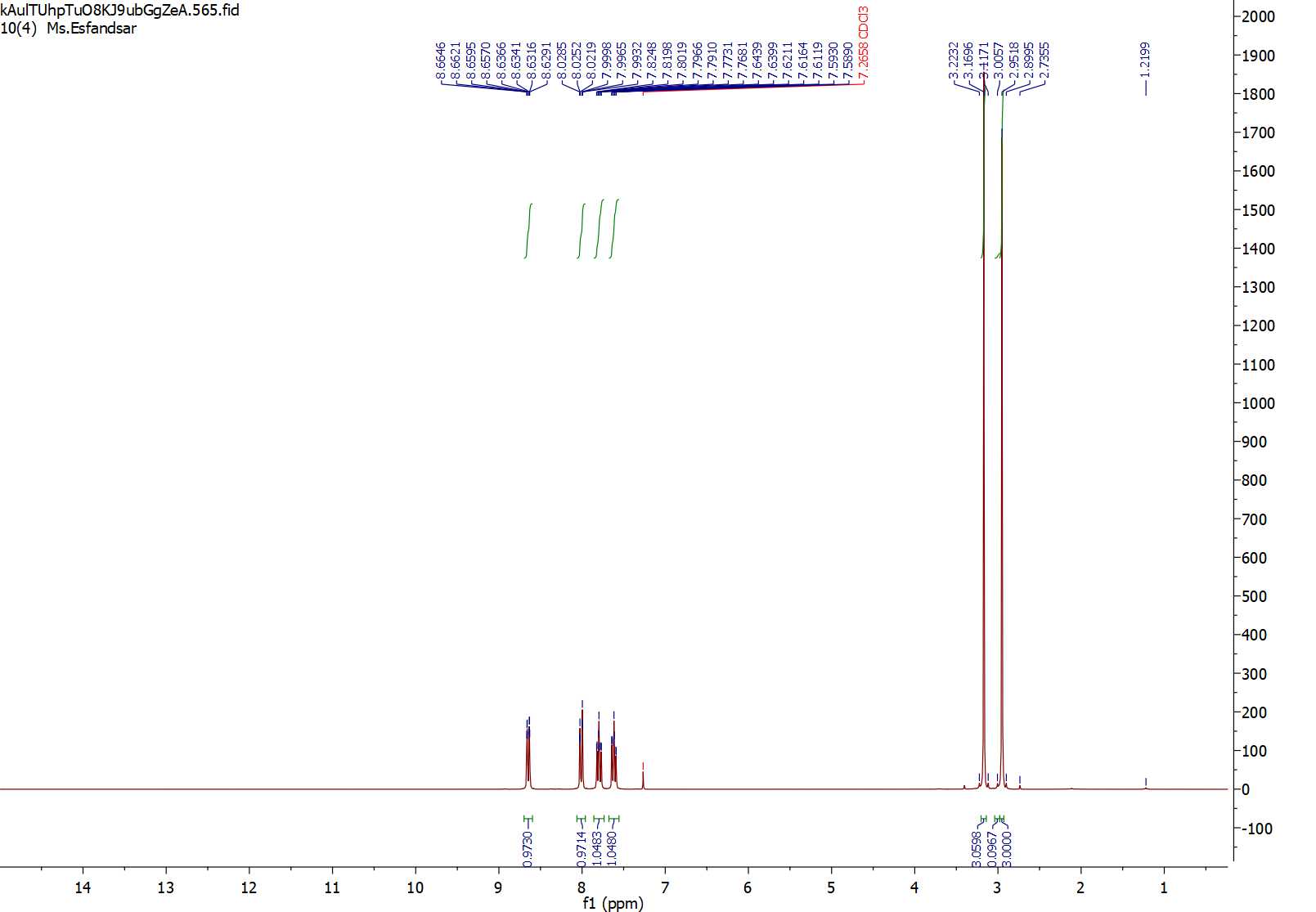


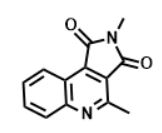


^13^C NMR of **7e**


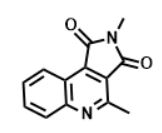

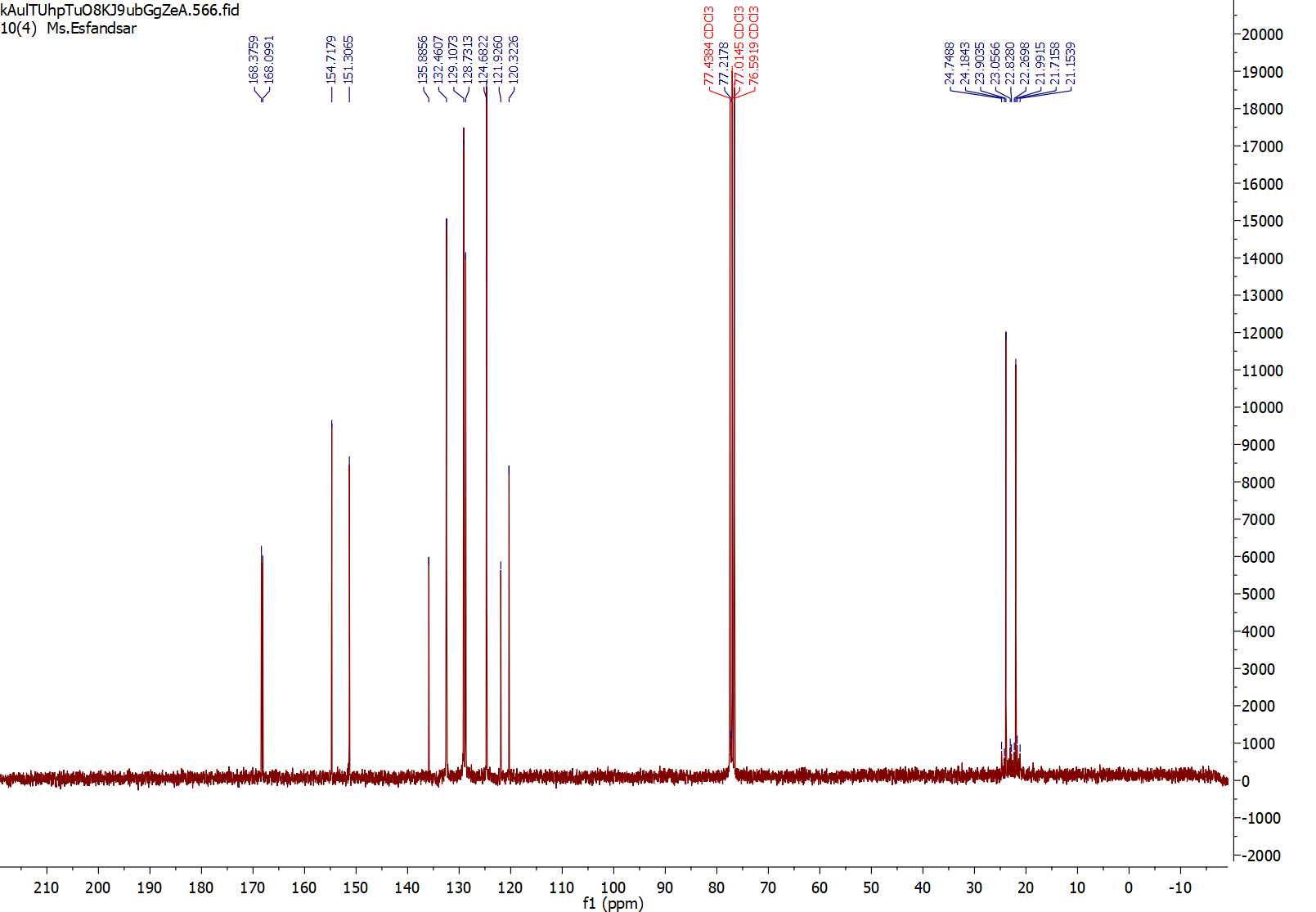


IR spectrum of **7e**


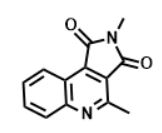


^1^H NMR of **7f**


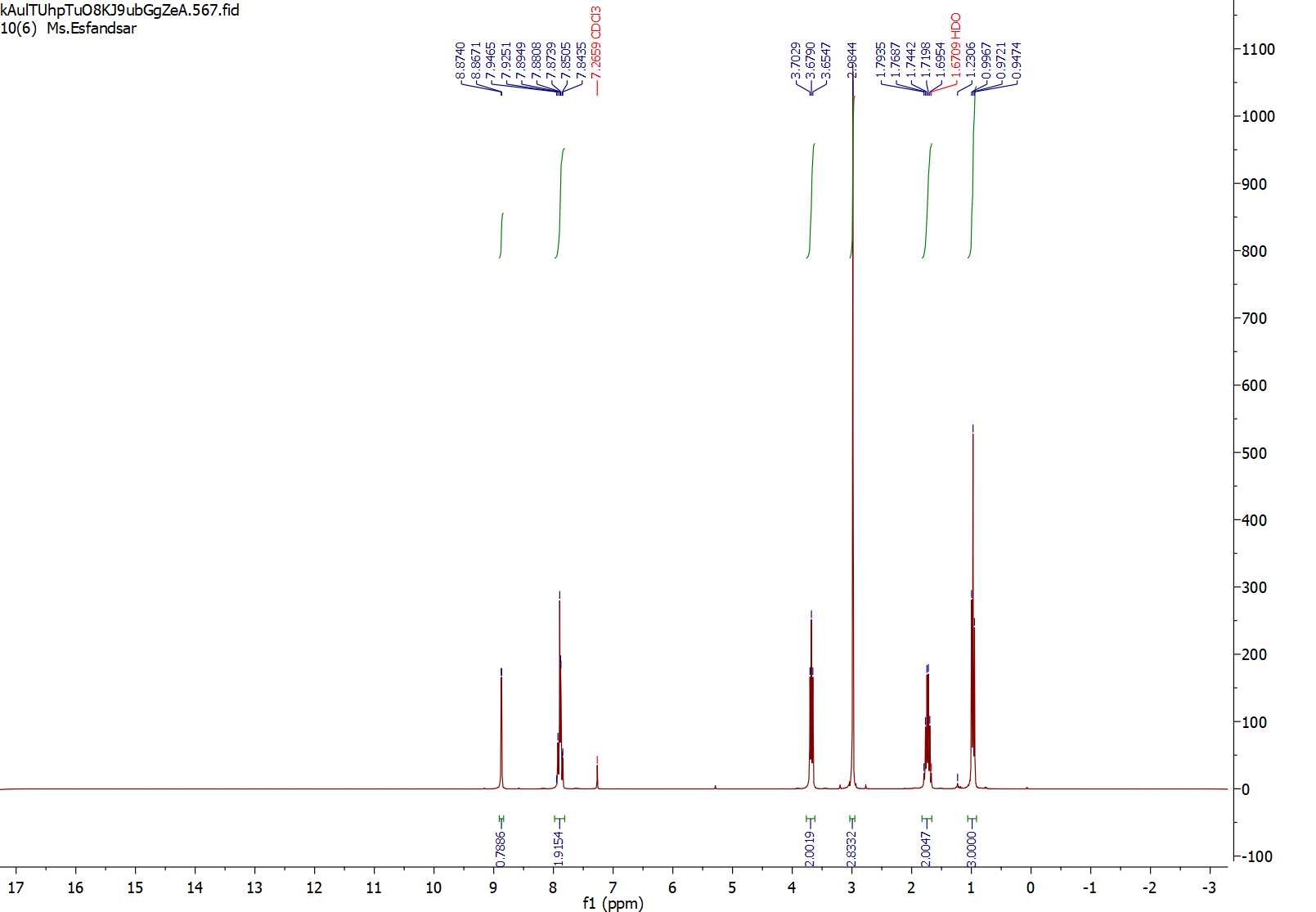


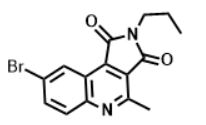


^13^C NMR of **7f**


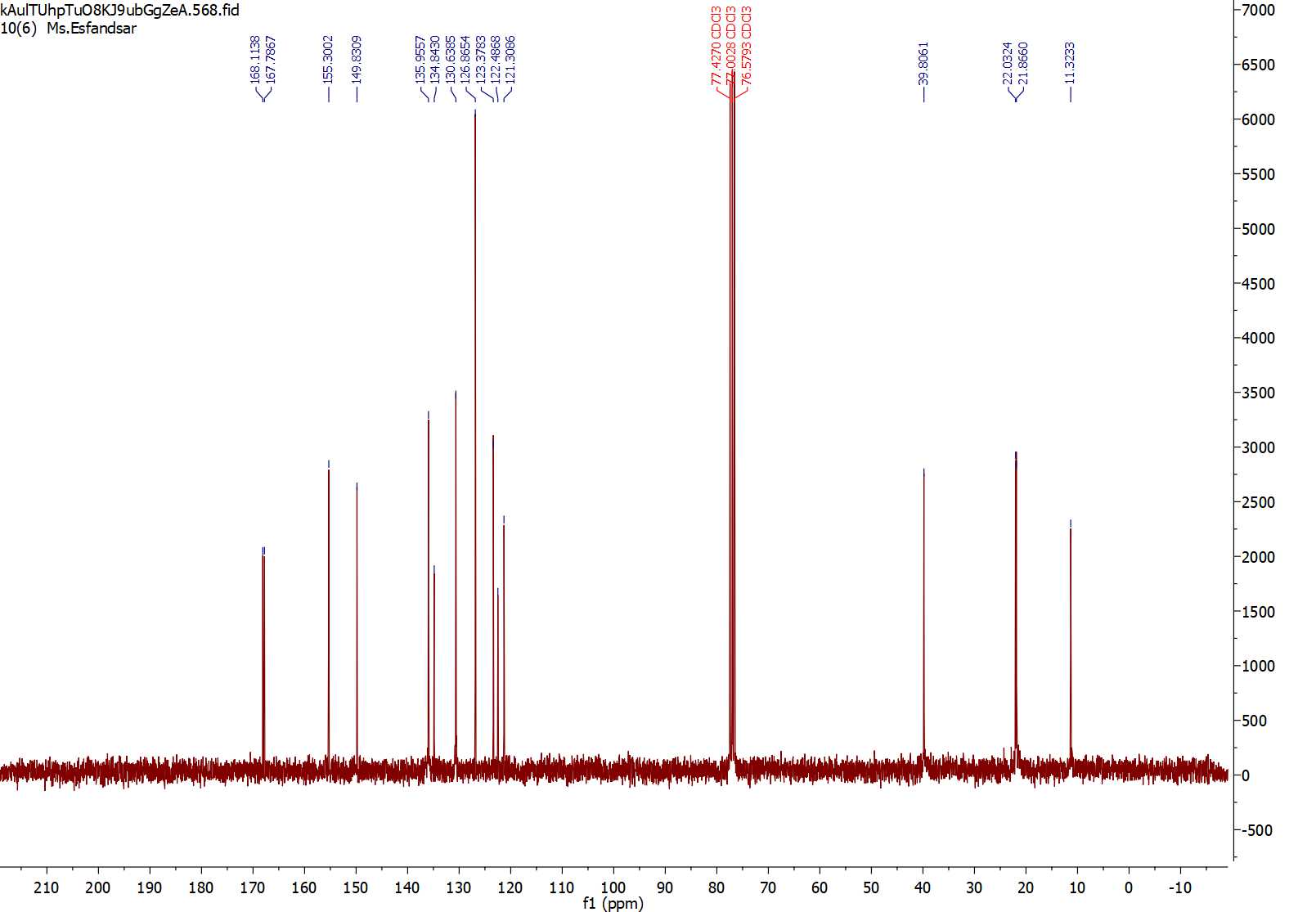


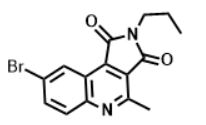


IR spectrum of **7f**

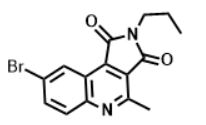


^1^H NMR of **7g**


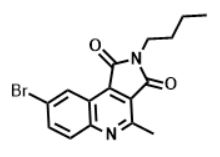

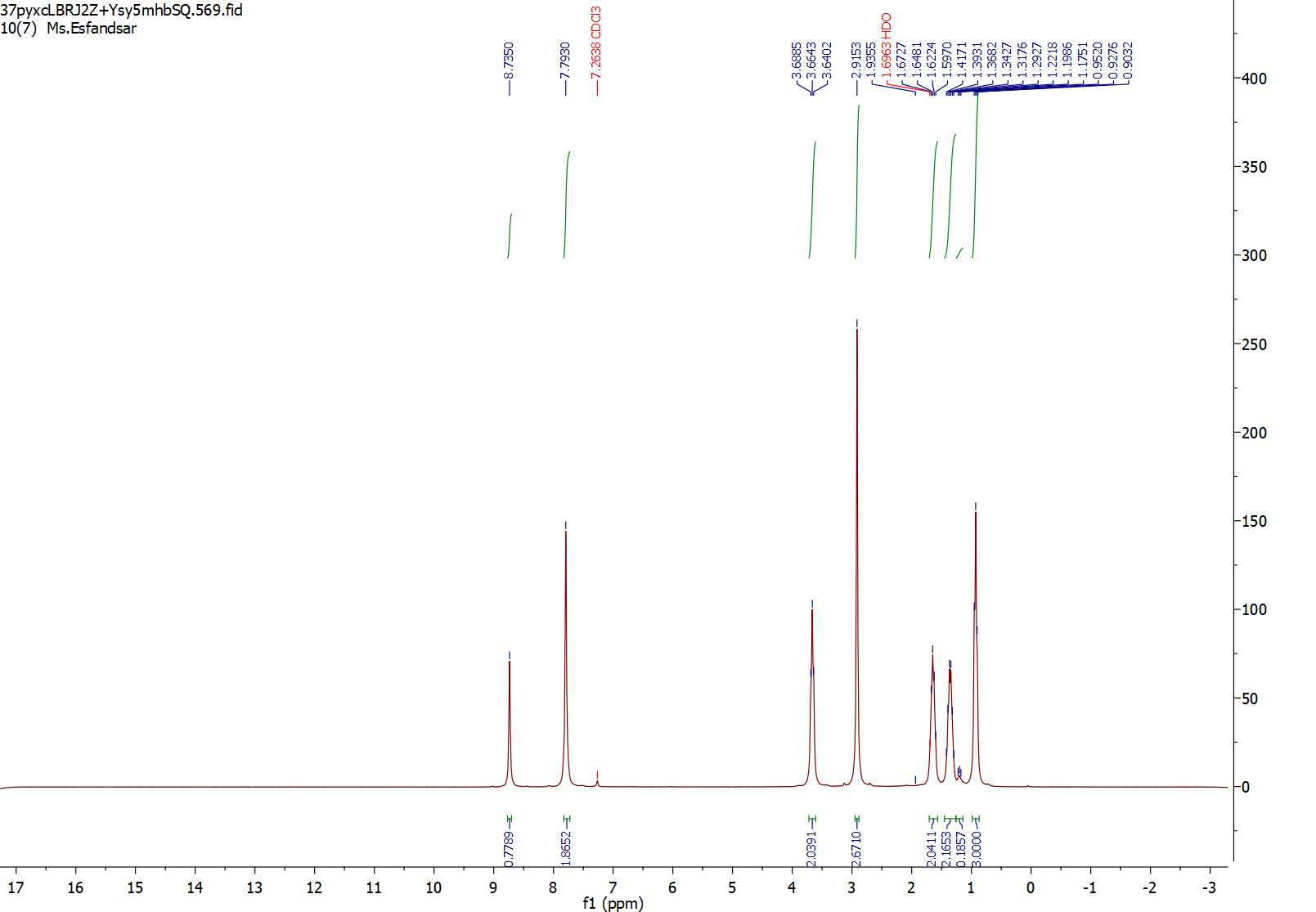


^13^C NMR of **7g**


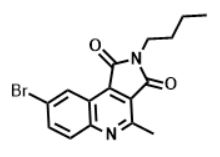

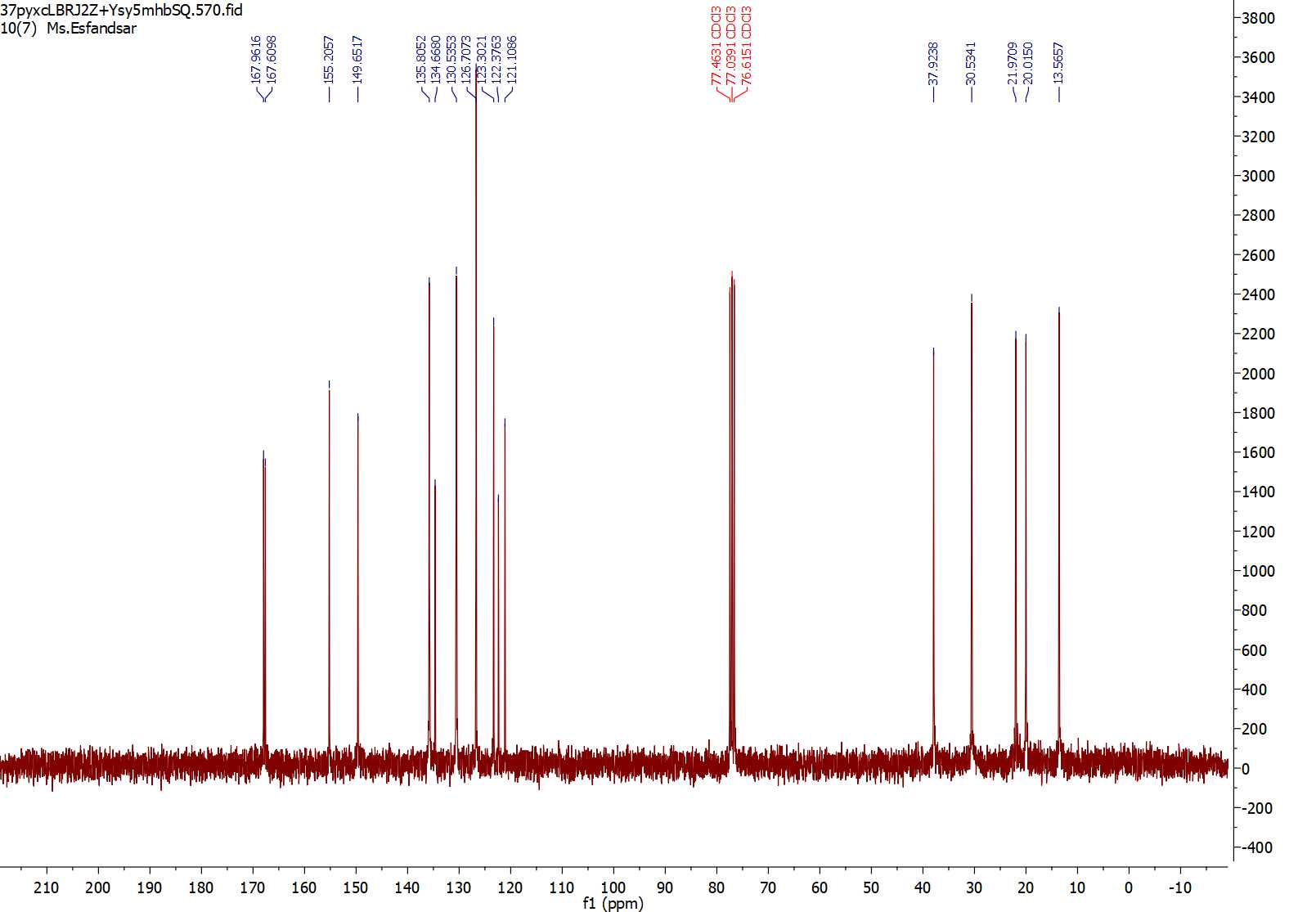


IR spectrum of **7g**

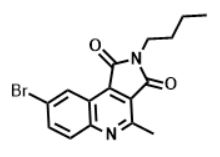


^1^H NMR of **7h**


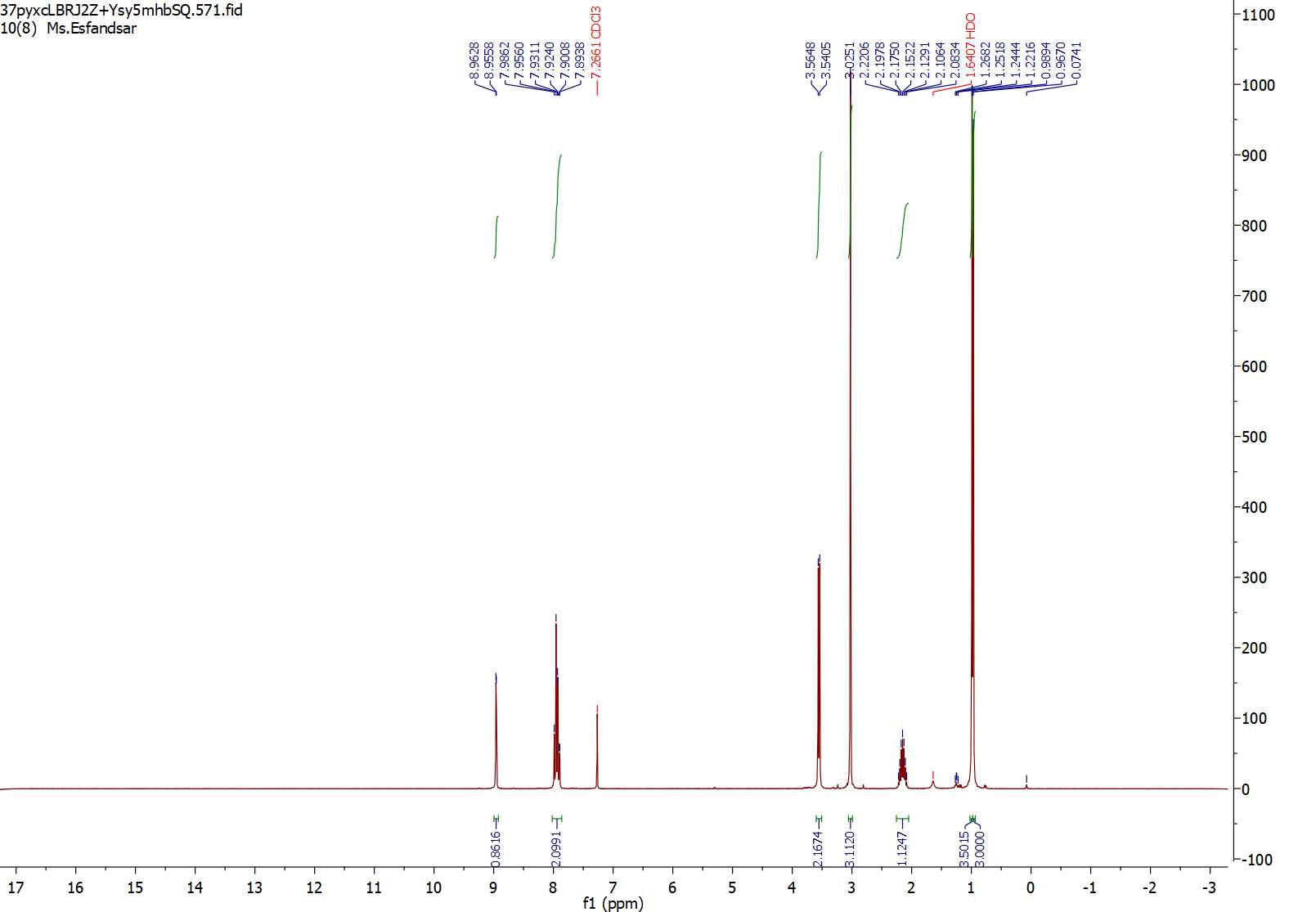


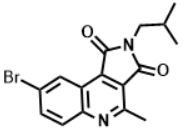


^13^C NMR of **7h**


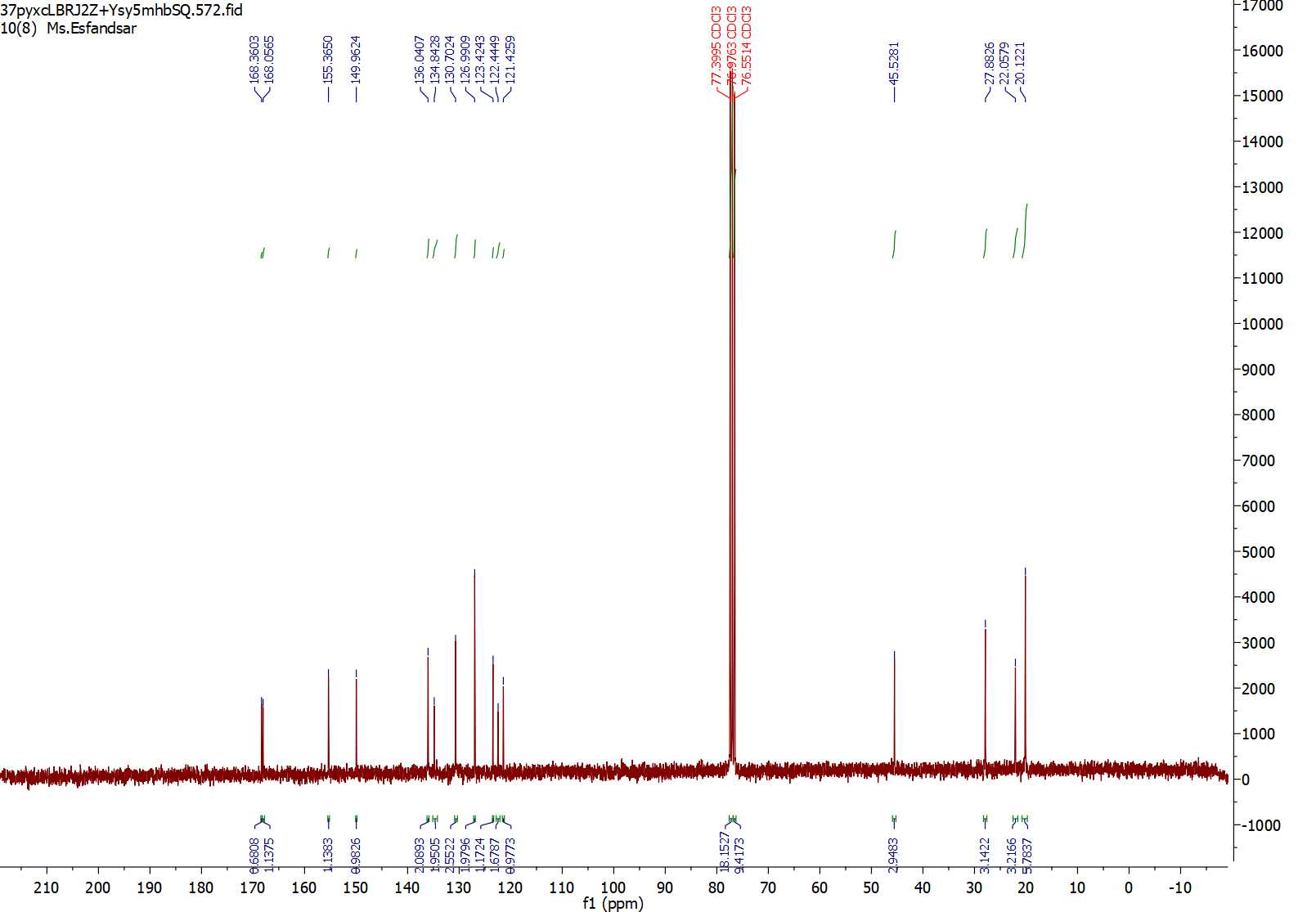


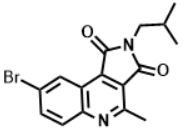


IR spectrum of **7h**

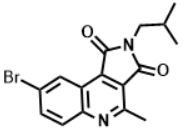

Supplement: Supplementary file 1 [file DataSheet1.docx]
